# Morphological and Molecular Changes in Juvenile Normal Human Fibroblasts Exposed to Simulated Microgravity

**DOI:** 10.1038/s41598-019-48378-9

**Published:** 2019-08-15

**Authors:** Christoph Buken, Jayashree Sahana, Thomas J. Corydon, Daniela Melnik, Johann Bauer, Markus Wehland, Marcus Krüger, Silke Balk, Nauras Abuagela, Manfred Infanger, Daniela Grimm

**Affiliations:** 1 Clinic for Plastic, Aesthetic and Hand Surgery, Otto von Guericke University Magdeburg, Leipziger Str. 44, 39120 Magdeburg, Germany; 2 Department for Biomedicine, Aarhus University, Høegh-Guldbergsgade 10, DK-8000 Aarhus C, Denmark; 3 Department of Ophthalmology, Aarhus University Hospital, 8200 Aarhus N, Palle Juul-Jensens Boulevard 99, DK-8200 Aarhus N, Denmark; 4 Max Planck Institute of Biochemistry, Am Klopferspitz 18, 82152 Planegg, Germany; 5 Gravitational Biology and Translational Regenerative Medicine, Faculty of Medicine and Mechanical Engineering, Otto von Guericke University Magdeburg, 39120 Magdeburg, Germany

**Keywords:** Extracellular matrix, Extracellular matrix, Growth factor signalling, Growth factor signalling, Molecular medicine

## Abstract

The literature suggests morphological alterations and molecular biological changes within the cellular milieu of human cells, exposed to microgravity (µ*g*), as many cell types assemble to multicellular spheroids (MCS). In this study we investigated juvenile normal human dermal fibroblasts (NHDF) grown in simulated µ*g* (s-µ*g*) on a random positioning machine (RPM), aiming to study changes in cell morphology, cytoskeleton, extracellular matrix (ECM), focal adhesion and growth factors. On the RPM, NHDF formed an adherent monolayer and compact MCS. For the two cell populations we found a differential regulation of fibronectin, laminin, collagen-IV, aggrecan, osteopontin, TIMP-1, integrin-β_1_, caveolin-1, E-cadherin, talin-1, vimentin, α-SM actin, TGF-β_1_, IL-8, MCP-1, MMP-1, and MMP-14 both on the transcriptional and/or translational level. Immunofluorescence staining revealed only slight structural changes in cytoskeletal components. Flow cytometry showed various membrane-bound proteins with considerable variations. *In silico* analyses of the regulated proteins revealed an interaction network, contributing to MCS growth via signals mediated by integrin-β_1_, E-cadherin, caveolin-1 and talin-1. In conclusion, s-µ*g*-conditions induced changes in the cytoskeleton, ECM, focal adhesion and growth behavior of NHDF and we identified for the first time factors involved in fibroblast 3D-assembly. This new knowledge might be of importance in tissue engineering, wound healing and cancer metastasis.

## Introduction

With recently growing ambitions in human spaceflight endeavors, the pathophysiologic mechanisms of many health problems that individuals in space regularly face, require further investigation. Astronauts in space often suffer from problems with the skin. The dermis of crew members frequently develops extensive allergy-like rashes, a fluid shift, slowed wound healing, and an alteration in skin physiological properties^1^. In the epidermis, a significant thinning of this skin layer has been reported in a mouse model in space, lowering the mechanical barrier against environmental forces, such as radiation^2^. In the dermal skin layer, the composition and ratio of collagen and elastin have been shown to be altered after a six-month space mission of ISS-astronauts, thus decreasing the skin’s overall elasticity^3^. These findings strongly suggest that morphology and structure of the skin are altered by microgravity (µ*g*). For this reason, the effects of µ*g* on dermal cells need to be characterized. As the key regulatory cell type of dermal integrity, we focused on juvenile normal human dermal fibroblasts (NHDF) and their reaction to an extended period of simulated microgravity (s-µ*g*), created by a random positioning machine (RPM).

Most fibroblasts are of mesenchymal origin and represent the major cell type of the connective tissue. An important role of the fibroblasts is to synthesize the components of the extracellular matrix (ECM) and maintain the ECM homeostasis. They produce the structural network for mammalian tissues and are involved in wound repair^4,5^. Fibroblasts can acquire an activated and immunoregulatory phenotype^6^. Moreover, fibroblasts play an important role in biological processes like inflammation, angiogenesis, cancer progression, as well as in tissue fibrosis^6^.

The first aim of this study is to focus on the morphology and the growth behavior of fibroblasts exposed to the RPM. Since several years, we have studied the behavior of benign and malignant cells with emphasis on proliferation, cellular differentiation, migration, apoptosis or with focus on the cytoskeleton, focal adhesion and ECM^7^. In this context we exposed human cells of various types to s-µ*g* and real microgravity (r-µ*g*)^8–13^. Normally, most cell types grow as a two-dimensional (2D) monolayer *in vitro*, when they are cultured in culture flasks and incubated in a standard cell culture incubator at 1*g* normal laboratory conditions on Earth. The cells leaving the monolayers in µ*g* assemble in a scaffold-free manner to three-dimensional (3D) multicellular spheroids (MCS) or to tubular structures, depending on the cell type^8–13^. This change in phenotype occurs during spaceflights as well as during exposure to µ*g*-simulators^14–16^. It is accompanied by alterations of the cytoskeletal network as well as by changes in the gene expression, protein synthesis and secretion patterns^7,17^.

The second aim of this study is to study possible changes in the ECM (laminin, fibronectin, proteoglycans, osteopontin), the cytoskeleton (actin, tubulin, vimentin), and focal adhesion (FA) factors (vinculin, talin-1, E-cadherin, focal adhesion kinase 1) of NHDF exposed to the RPM. It is known that fibronectin is involved in spheroid formation of thyroid cancer cells^18^.

It has been found earlier that spheroids generated from periodontal fibroblasts using the liquid overlay technique were able to adhere to biological membranes and might be used as an adjunct for periodontal regeneration^19^. Moreover, fibroblasts are often used for co-cultures with cancer cells as a model for epithelial–mesenchymal transition *in vitro*^20^. As a major component of the tumor microenvironment, cancer-associated fibroblasts are involved in cancer progression and drug resistance^21^. For these studies often scaffolds are used. Growing 3D constructs in space or using µ*g* provided by the RPM as a technology for tissue-engineering (TE) makes it possible to work without any scaffolds. This type of MCS has to be characterized. Therefore, it is necessary to investigate structure, morphology, ECM, cytoskeleton together with FA factors in NHDF in more detail.

Another objective is to investigate expectable changes in growth factors (connective tissue growth factor (CTGF), epidermal growth factor (EGF), vascular endothelial growth factor (VEGF), and cytokines (interleukin-6 (IL-6), interleukin 8 (IL-8). In addition, we focused on changes in the productive potential of fibroblasts by investigating their collagen synthesis (collagen type I, III and IV) and metabolism (tissue inhibitor of metalloproteinases 1 (TIMP1), matrix metalloproteinases (MMPs)). We studied these factors because some of them are known to promote growth of human cells. CTGF and EGF had shown to be involved in spheroid formation of thyroid cancer cells in space^16^. VEGF is known to be involved in 3D growth of endothelial cells^22^ and the cytokines IL-6 and IL-8 improved spheroid formation of thyroid cancer cells under 1*g*-conditions using the liquid-overlay technique to engineer MCS^23^.

Finally, we investigated possible interactions of the genes and proteins determined in NHDF exposed to the RPM and performed a pathway analysis.

## Results and Discussion

### Characterization and morphology of juvenile normal human dermal fibroblasts exposed to the RPM

**Figure 1:**
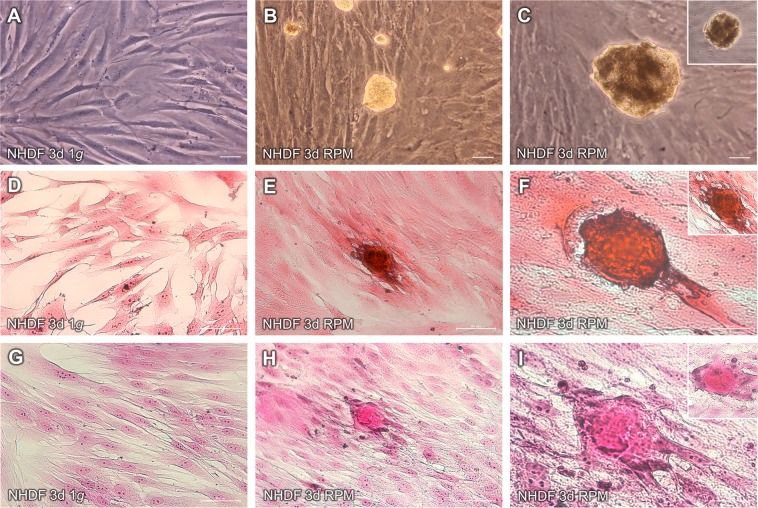
Characterization of morphological alterations in juvenile NHDF cells after a three-day RPM-exposure. Phase contrast microscopy images of 1*g*-control cells (**A**), RPM-AD (**B**) and RPM-MCS (**C**). Hematoxylin-Eosin staining of 1*g*-control cells (**D**), RPM-AD (**E**) and RPM-MCS (**F**). Elastica van Gieson staining of 1*g*-control cells (**G**), RPM-AD (**H**) and RPM-MCS (**I**). Scale bars: 50 µm.

A main objective of this study was to examine structural alterations in juvenile NHDF exposed for three days (3d) to the RPM. We studied the morphology of adherent cells and of 3D MCS. Phase contrast microscopy showed that the NHDF cultured under static 1*g*-conditions grew as a normal two-dimensional (2D) monolayer, when the flasks were placed next to the RPM in a commercially available incubator (Fig. 1A). The cells revealed a spindle-shaped morphology. They were disjointed and scattered, when covering the cell culture flask bottom. However, after a three-day exposure of NHDF to the RPM, two phenotypes were found: one part of the fibroblasts grew as 3D MCS and the other one remained adherently and grew as a 2D monolayer (Fig. 1B,C). Both cellular phenotypes were investigated by qPCR, Western blotting, and immunofluorescence staining (IFS). The adherent cells were further studied by flow cytometry. Multianalyte Profiling (MAP) technology was used to measure the cytokine levels in the cell culture supernatants.

MCS formation after exposure to conditions of s-µ*g* had been repeatedly reported with various cell types, such as EA.hy926 endothelial cells, normal thyroid cells (Nthy-ori 3-1), FTC-133 follicular thyroid cancer cells and MCF-7 breast cancer cells^8–13^. Studies with NHDF exposed to a RPM can increase the current knowledge in TE. The RPM is an interesting device widely used for TE^24–26^. Fibroblasts can be used for co-culture experiments in order to e.g. trigger formation of vessels and other tissues and thus, it is important to know whether fibroblasts can grow as 3D MCS or remain growing adherently in the cell culture flasks.

In addition, future co-culture models of fibroblasts, endothelial cells and cancer cells using the RPM may allow a further understanding of metastasis and tumor progression. The interaction among heterotypic fibroblasts and cancer cells contributes to cancer progression. Therefore, understanding its complex microenvironment is important. NHDF can be used in co-cultures with various malignant cell types. Today MCS are cultured to examine the molecular mechanisms involved in tumorigenesis, cancer biology, angiogenesis and for drug testing of e.g. chemotherapeutic agents or tyrosine kinase inhibitors. In addition, MCS are studied in toxicology and radiation biology. Clarifying the mechanisms of µ*g*-dependent changes is an up-to-date topic for increasing the current knowledge in space medicine and developing new therapeutical options or countermeasures that can be translated to *in vivo* models, while sparing laboratory animals. In this regard, the science areas TE, cancer research and pharmacology merge smoothly.

As mentioned above, we observed that in the s-µ*g*-group one part of the cells grew in MCS floating in the medium, while another part grew in MCS that were still in connection with the adherent cell monolayer (AD). Hematoxylin-Eosin (HE; Fig. 1D–F) and Elastica van Gieson (EvG; Fig. 1G–I) staining showed these MCS (Fig. 1E,F,H,I), which were not observed in the 1*g*-control cells (Fig. 1D,G). Additionally, there was a group of pre-MCS (Fig. 1B). These initially unicellular configurations resemble an intermediate stage, which is still adherent to the bottom of the culture flask and will develop into fully developed MCS. Cell culture flasks of juvenile fibroblasts generally showed a large number of MCS after a 3-day RPM-exposure. An intermediate type was also detectable in endothelial cells (EC) exposed to an RPM. The EC grew either within a monolayer adhering to the culture flask bottom, or within an elongated cellular aggregate floating in the culture medium or within an assembly of cells consisting mainly of two cell rows still in contact with the plastic dish surface^27^. These double-row EC assemblies were considered to be progenitors of the elongated cylindrical structures (called tubular structures) tissue engineered on the RPM^27^.

Also in cancer cell and EC lines, AD and MCS cells were found in each culture flask mounted on the RPM. This raised the question, why individual cells of one population behave differently. Until now, preliminary hints were observed, which could explain the different behavior of individual cells of a given population. Flow cytometric and electrophoretic studies revealed a considerable quantitative variation of distinct surface related biochemical components between the individual cells of a thyroid cancer cell line^28,29^. In addition, ASAP1, a protein whose overexpression destabilizes the interaction of focal adhesion kinase 1 and paxillin^30^ was found only in FTC-133 MCS cells^31^. SRC that loosens E-cadherin-E-cadherin binding within the tight junctions by phosphorylation was lacking in MCF-7 AD cells^11^. Furthermore, FTC-133 follicular thyroid cancer cells formed more and larger spheroids during 3 days of culturing on the RPM than CGTH W-1 cells. By a proteome analysis of the two cell populations integrin-α5 and myosin-10 were only detected in the FTC-133 cells. Hence, currently, one may assume that some key proteins, responsible for spheroid formation, reach a critical accumulation threshold only in a part of the cells of a population. Therefore, we included flow cytometry in this study as an additional tool to quantify proteins.

### Impact of simulated microgravity on extracellular matrix proteins, the cytoskeleton, and focal adhesion factors

**Figure 2:**
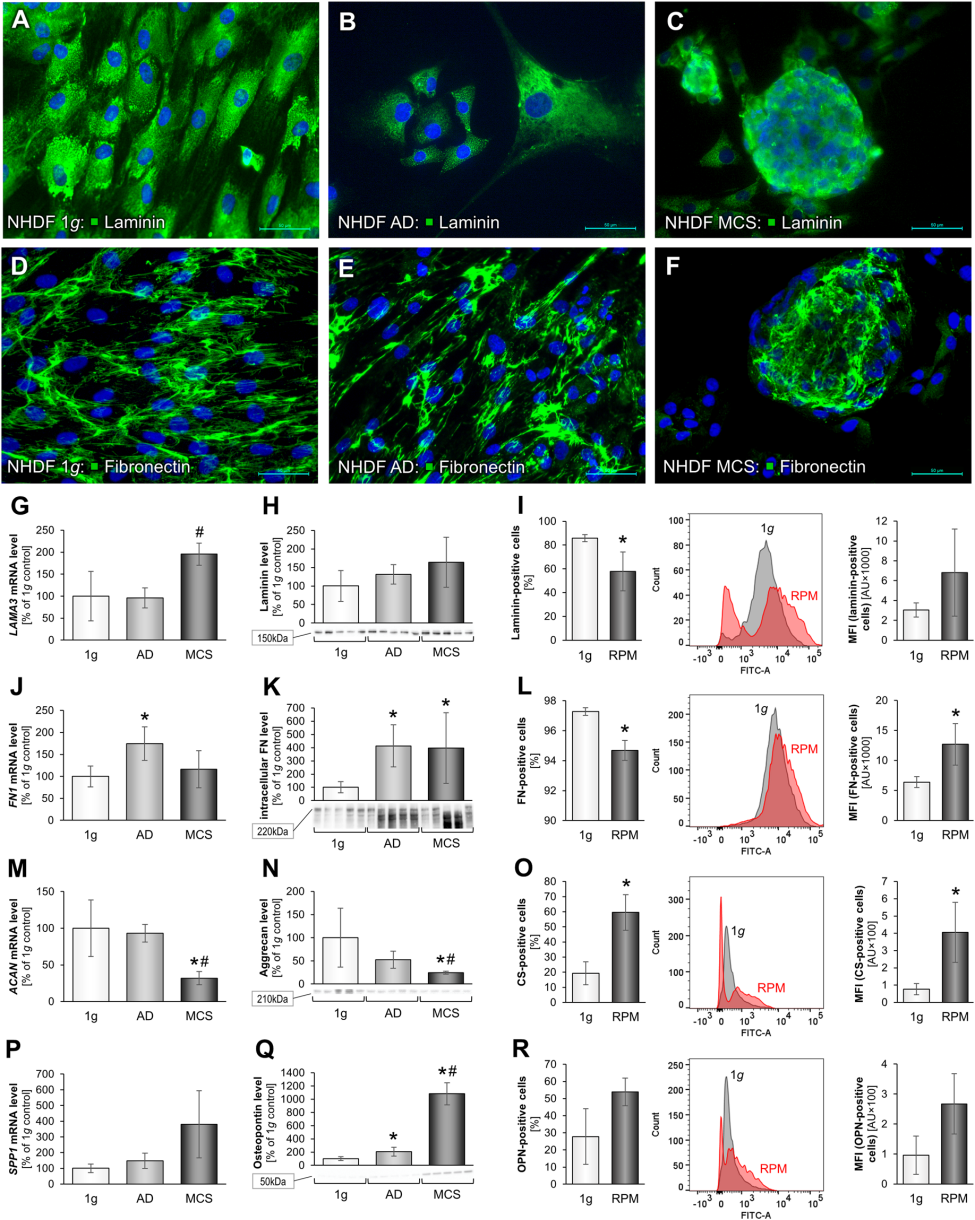
Changes in the ECM composition of NHDF after a three-day RPM-exposure: Immunofluorescence images of 1*g*-control cells, RPM-AD and RPM-MCS of laminin (**A**–**C**) and fibronectin (**D**–**F**). Transcriptional and translational laminin analysis: Quantitative gene expression levels of *LAMA3* (**G**), intracellular laminin levels (**H**) and flow cytometric analysis of laminin-labeled cells (**I**) displaying the percentage of laminin-positive cells as well as alteration of the median fluorescence intensity (MFI). Transcriptional and translational fibronectin analysis: Quantitative gene expression levels of *FN1* (**J**), intracellular fibronectin levels (**K**) and flow cytometric analysis of fibronectin-labeled cells (**L**). Transcriptional and translational aggrecan analysis: Quantitative gene expression level of *ACAN* (**M**), intracellular aggrecan levels (**N**) and flow cytometric analysis of chondroitin sulfate-labeled cells (**O**). Transcriptional and translational osteopontin analysis: Quantitative gene expression levels of *SPP1* (**P**), intracellular osteopontin levels (**Q**) and flow cytometric analysis of osteopontin-labeled cells (**R**). Full-length blots of cropped Western blot images are presented in Supplementary Fig. S1. *p < 0.05 1*g* vs. RPM; ^#^p < 0.05 AD vs. MCS. Scale bars: 50 µm.

In a next step, we first focused on the ECM proteins laminin, fibronectin, aggrecan, chondroitin sulfate and osteopontin. The ECM plays a key role in cell adhesion, migration, proliferation and growth and may be altered by gravitational unloading. The ECM is responsible for the structure of the tissues. Three groups of samples were collected at day 3: 1*g*-control group, RPM-AD group and RPM-MCS group. To investigate possible changes in the ECM composition after a 3-day RPM-exposure, we performed IFS of each group and investigated laminin (Fig. 2A–C) and fibronectin (Fig. 2D–F).

While a quantitative analysis of fluorescence intensities for laminin and all other IFS pictures was not made due to the fact that the 3D nature of the MCS results in a cumulative fluorescence signal caused by multiple cell layers, thus most certainly causing artifactual measurements, qualitative changes were assessable. Compared to the 1*g*-control, RPM-adherent (AD) cells seemed to exhibit a slightly smaller ratio of fibrous vs. granular laminin-positive structures. In addition, the fibres in AD cells were less organized (Fig. 2B). The highly packed MCS did not allow for a more detailed examination of laminin structures (Fig. 2C). In qPCR analyses we found a significant *LAMA3* mRNA increase in the MCS group (Fig. 2G). Western blotting revealed a slight increase in MCS, which was not significant compared to 1*g*-controls (Fig. 2H). The amount of laminin-positive AD cells was reduced, but they exerted a higher median fluorescence intensity (MFI) as determined by flow cytometry (Fig. 2I). Laminin and fibronectin were expressed in a lower number of cells, but laminin- and fibronectin-positive cells showed higher average protein levels as reflected by MFI (Fig. 2I,L).

Laminin is a glycoprotein and a substantial part of the basal laminae^32^. It is also a cell adhesion molecule and it became apparent that laminin is a gravi-sensitive protein. Changes in the amount of laminin in MCS had also been detected in earlier studies investigating cardiac fibroblasts, follicular thyroid cancer cells or endothelial cells after RPM-exposure^22,33,34^. These results are cell type-specific. Human adult retinal epithelium (ARPE-19) cells revealed a significant reduction of *LAMB2* mRNA and laminin protein after a 5-day and 10-day RPM-exposure^17^.

The visualization of fibronectin by IFS showed no discernable differences in fibronectin structure and distribution between 1*g*-controls (Fig. 2D), AD cells (Fig. 2E), and MCS (Fig. 2F). qPCR analyses, revealed a significant 1.5-fold increase in the *FN1* gene expression in AD cells (Fig. 2J). Fibronectin is the main mediator of cell-ECM interaction^35^ and an important factor involved in early wound repair^36,37^. In addition, the corresponding protein was elevated in RPM-AD and RPM-MCS (Fig. 2K). The number of fibronectin-positive RPM-AD cells was reduced compared to 1*g*-cells, but they exerted a significant higher MFI (Fig. 2L).

We further focused on the gene expression of the ECM components aggrecan (*ACAN*) and osteopontin (secreted phosphoprotein 1 (*SPP1*)). qPCR analyses revealed a significant decrease in the *ACAN* gene expression in MCS as compared to the other groups (Fig. 2M). This observation was coherent with the Western blot data (Fig. 2N). We also studied chondroitin sulfate (CS) by flow cytometric analysis and found an increase in CS-positive cells on the RPM (Fig. 2O). The proteoglycan aggrecan, best known for its water binding capacity via hydrated gel structures in hyaline- and fibrocartilage^38^, acts as chondro-protective as well as anti-inflammatory agent and inhibitor of chondrocyte apoptosis^39^. Interestingly, this finding was reciprocal, when investigating osteopontin. Although not significant in qPCR data, the *SPP1* gene expression was almost 4-fold elevated in MCS cells as compared to 1*g*-control cells (Fig. 2P). In addition, the osteopontin protein was significantly elevated in MCS compared to the other groups (Fig. 2Q). We were further able to confirm this data with the results from flow cytometry (Fig. 2R). This supports our findings from previous studies^22^.

Taken together, the enhancement of fibronectin proteins in MCS is no novel finding and is comparable with earlier results deriving from the TE of preliminary vessels^40^. Increases in fibronectin were also found in tube-like structures of EC after a 35-day RPM-exposure^41^. In addition, 3D human hair dermal papilla spheroids revealed an increase in ECM proteins compared to 1*g*-cultures^42^. Similar results were measured in MCS when thyroid cancer spheroids exposed to an RPM were investigated^43,44^. A key role of fibronectin is indicated for all phases of wound healing^36^. Furthermore, an enhanced fibronectin mRNA is detectable in abnormal wound healing when keloids are formed^45^. Astronauts suffer of an abnormal wound healing and thus, it is important to increase the current knowledge about the changes in fibroblasts cultured in µ*g* in regard to their ECM synthesis^35^. ECM proteins, adhesion molecules, and cytoskeletal proteins form a dynamic network interacting with signalling molecules as an adaptive response to µ*g*.

**Figure 3:**
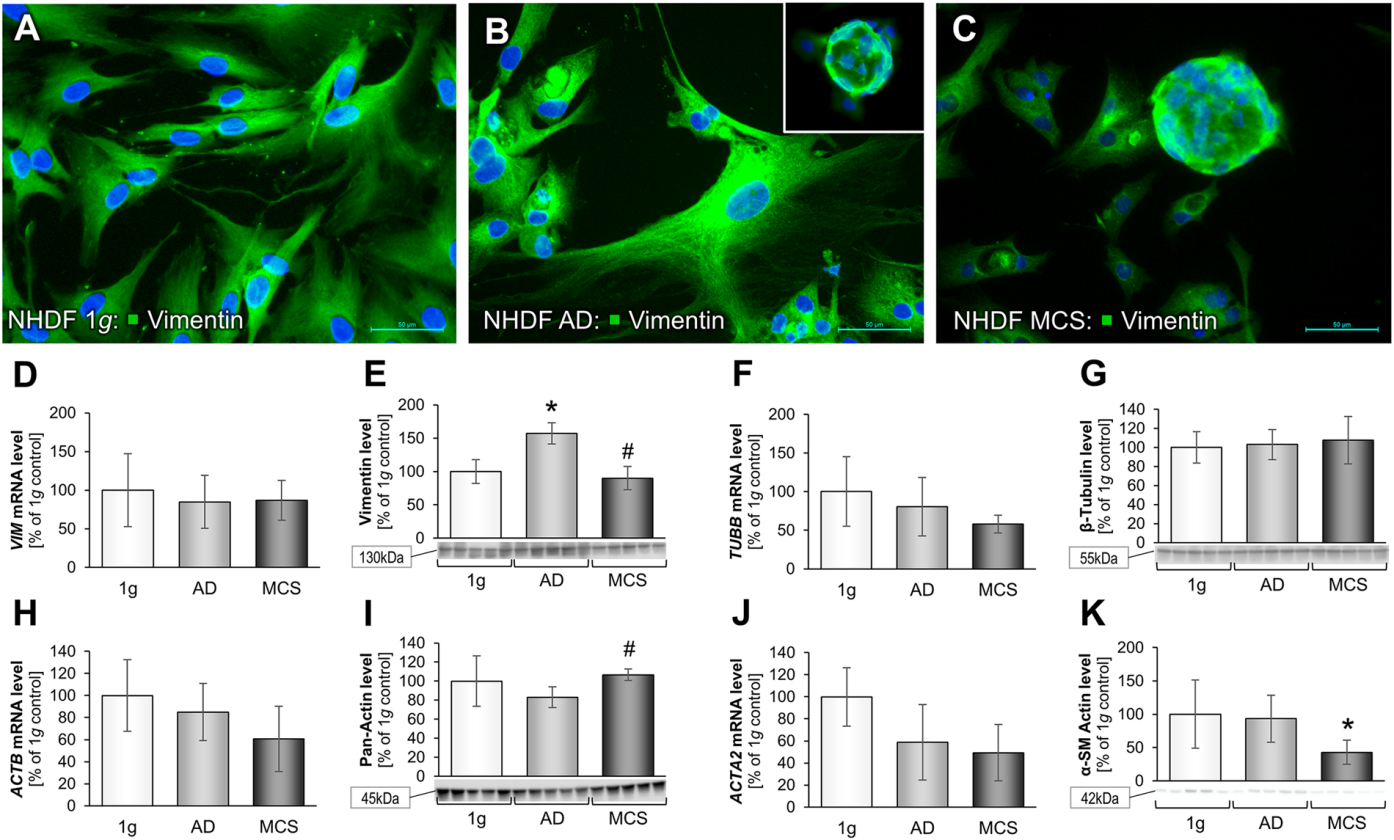
Cytoskeletal changes in NHDF cells after a 3-day s-µ*g*-exposure: Immunofluorescence images of 1*g*-control cells, RPM-AD and RPM-MCS of vimentin (**A**–**C**). Transcriptional and translational vimentin analysis: Quantitative gene expression levels of *VIM* (**D**) and intracellular vimentin levels (**E**). Transcriptional and translational β-tubulin analysis: Quantitative gene expression levels of *TUBB* (**F**) and intracellular β-tubulin levels (**G**). Quantitative gene expression levels of *ACTB* (**H**). Intracellular pan-actin levels (**I**). Transcriptional and translational alpha smooth muscle actin analysis: Quantitative gene expression levels of *ACTA2* (**J**) and intracellular alpha smooth muscle actin levels (**K**). Full-length blots of cropped Western blot images are presented in Supplementary Fig. S2. *p < 0.05 1*g* vs. RPM; ^#^p < 0.05 AD vs. MCS. Scale bars: 50 µm.

Therefore, secondly, we investigated the cytoskeletal factors vimentin, tubulin and actin in NHDF exposed to the RPM. IFS of the intermediate filament vimentin was performed to further characterize the morphological changes under s-µ*g*-influence with regard to the cytoskeleton, where we found no differences between 1*g*-controls, AD and MCS (Fig. 3A–C). The corresponding *VIM* gene expression was not changed in all groups (Fig. 3D). On the other hand, we found a highly significant increase of the RPM-AD protein content in comparison to the other groups (Fig. 3E). The occurrence of vimentin in NHDF cells is evident by their mesenchymal origin. As a type-3 intermediate filament it plays a major role in establishing the cytoskeletal framework of the cell^46^. In recent breast cancer cell studies vimentin was described as a mediator of cytoskeletal organization in epithelial to mesenchymal transition in metastasis, where it plays a role in altering cell shape and allowing motility and adhesion^47^. It is known that fibroblasts and other benign cells are used by a developing cancer to form its own tumor-specific stroma that is afterwards able to influence cancer progression and metastasis^48^. These cancer-associated fibroblasts (CAFs) can be obtained by the activation of normal fibroblasts with cancer cell conditioned fibroblast growth media using breast cancer cells or other cancer types. Activated human primary mammary fibroblasts HPMFs exerted an upregulation of vimentin and desmin among others^48^.

CAFs will be investigated scaffold-free on an RPM to study the influence of µ*g* on these cells and were used for co-culture experiments. These studies will be performed in the future.

To further investigate the impact of an RPM-exposure on the cytoskeleton, we performed qPCR and Western blot analyses of various other components of the cytoskeleton. Investigating *TUBB* gene expression and its β-tubulin protein no significantly change was detectable (Fig. 3F,G).

In addition, we focused on the actin gene expression and protein content. Actin is a dynamic structural protein and microfilament with a diameter of about 7 nm, and is responsible for the stability of the cell, cell movement and among others for the formation of microvilli, pseudopodia, adherence junctions and tight junctions. First, we investigated the *ACTB* gene expression, which was not significantly changed (Fig. 3H). Pan-actin protein was also not changed in MCS compared to 1*g*-controls (Fig. 3I). There was a slight decrease in the alpha smooth muscle actin gene (*ACTA2*) in AD and MCS samples compared to 1*g*-control samples (Fig. 3J). Although the qPCR data did not show any significant changes, the Western blot results revealed a significant reduction in the MCS group compared to 1*g* (Fig. 3K). β-actin protein was not changed in all samples, and served as loading protein for the Western blot analyses. β-actin as major component of the cytoskeleton and β-tubulin as essential part of microtubules play major roles in the cytoskeletal integrity, cellular motility and migration^49–51^. This is a very interesting finding because these results demonstrate that the cytoskeletal genes and proteins are stable after a 3-day s-µ*g-*exposure of NHDF. This is noteworthy, as other cell types, such as chondrocytes, thyroid cancer cells or breast cancer cells exhibited more pronounced changes in the cytoskeletal conformation after exposure to both short- and long-term r- or s-µ*g*^38,46,52–54^. Moreover, earlier studies have shown that fibroblasts cultured in hypergravity of 20*g* for 8 days revealed only moderate cytoskeletal changes and that accelaration forces below 15*g* had no effect at all^55^. This is an indication that the extent of cellular changes in response to altered gravity conditions is cell type-specific and we speculate that fibroblasts are comparatively more resistant against this kind of mechanical challenge than other cells.

In summary, these observations are new and obviously the ECM and the cytoskeleton of fibroblasts are affected and altered in earlier phases by µ*g*. The cytoskeletal role in sensing gravitational changes was previously stated by the tensegrity model^56–59^. Subsequently, the cytoskeletal role on initiating cell signaling changes of NHDF under µ*g*-conditions, e.g. apoptotic behavior, has to be further investigated at earlier phases obtained during a parabolic flight campaign or sounding rocket mission.

Moreover, thirdly we focused on focal adhesions in NHDF exposed to the RPM. FAs, also called cell–matrix adhesions, are subcellular structures mediating cellular signaling in response to ECM adhesion and serve as mechanical linkages to the ECM. To further investigate the impact of a RPM-exposure to NHDF, we focused on FA proteins, as NHDF cells tend to grow three-dimensionally. FAs play an important role in this process, e.g. through sensing of the µ*g*-environment^60,61^. To further characterize alterations in FA proteins, we focused on vinculin as a major protein of interest. Vinculin is a protein with profound impact on cell-matrix adhesion and intercellular junctions and plays a major role in fibroblast-mediated wound healing^62^. Additionally, it has a vital role in mechanotransduction with integrins at focal adhesion sites^63^. Vinculin also directly interacts with talin, integrins and actin, hence allowing a proper cellular migration and orchestrating focal adhesion^64^. Vinculin is a component of the adherence junctions. It mediates cellular and extracellular signals.

**Figure 4:**
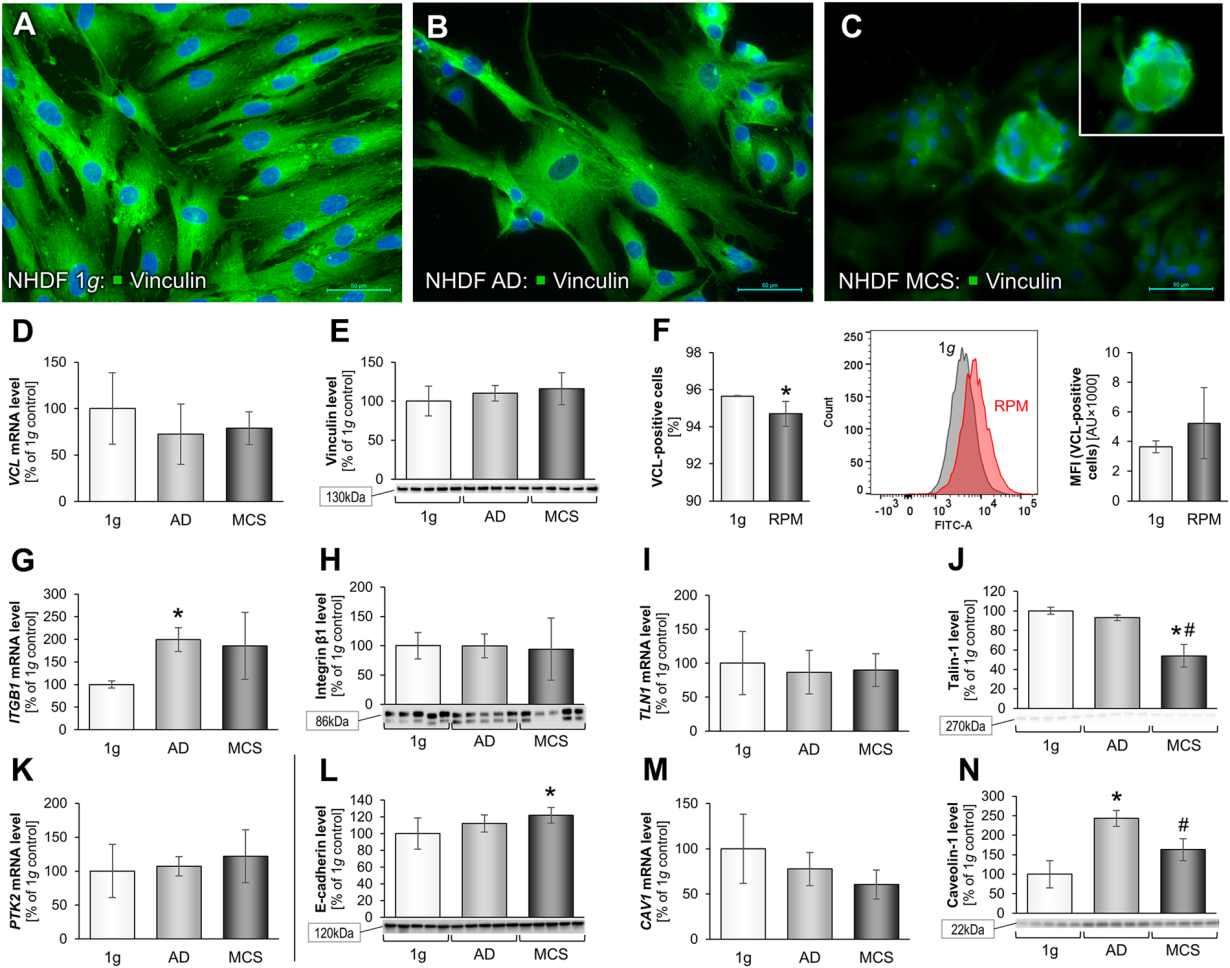
Alterations in focal adhesion-associated genes and proteins after a 3-day s-µ*g*-exposure: Immunofluorescence images of 1*g*-control cells, RPM-AD and RPM-MCS of vinculin (**A**–**C**). Transcriptional and translational vinculin analysis: Quantitative gene expression levels of *VCL* (**D**), intracellular vinculin levels (**E**) and flow cytometric analysis of vinculin-labeled cells (**F**). Transcriptional and translational integrin-β_1_ analysis: Quantitative gene expression levels of *ITGB1* (**G**) and intracellular integrin-β_1_ levels (**H**). Transcriptional and translational talin analysis: Quantitative gene expression levels of *TLN1* (**I**) and intracellular talin-1 levels (**J**). Quantitative gene expression levels of *FAK1*/*PTK2* (**K**). Intracellular E-cadherin levels (**L**). Transcriptional and translational caveolin-1 analysis: Quantitative gene expression levels of *CAV1* (**M**) and intracellular caveolin-1 levels (**N**). Full-length blots of cropped Western blot images are presented in Supplementary Fig. S3. *p < 0.05 1*g* vs. RPM; ^#^p < 0.05 AD vs. MCS. Scale bars: 50 µm.

Structural and morphological changes in vinculin were made visible by IFS (Fig. 4A–C). It became apparent, that there was a distinct reduction of the amount of micro-spiculae in the RPM-AD (Fig. 4B) and RPM-MCS groups (Fig. 4C), when comparing them to the 1*g*-control cells (Fig. 4A). Analysis of the *VCL* gene expression profile showed a tendency to decrease in the RPM- and AD-group (Fig. 4D). Furthermore, the protein content of vinculin was not changed in the MCS group (Fig. 4E). Flow cytometry reveled a significant reduction of vinculin in the RPM-AD group and an increase in MFI (Fig. 4F).

Another important component of the focal FAs is the cytosolic and mechanosensitive protein talin. It links integrin direct and indirect via vinculin to the cytoskeleton. Talin-1 plays a role in extravasation, cell adhesion, trans-endothelial migration and invasion. In addition, integrins bind to talin and talin binds to vinculin and thus they influence cell adhesion. The integrin receptors are involved in the attachment of adherent cells to the ECM. Talin connects the integrins to the actin cytoskeleton and acts as mechanotransductor. Our qPCR data for integrin-β_1_ (*ITGB1*) showed a highly significant increase of the *ITGB1* gene expression in the AD group compared to the 1*g*-control group. *ITGB1* was not changed in MCS (Fig. 4G). The corresponding integrin-β_1_ protein was not altered in all groups (Fig. 4H). The talin-1 (*TLN1*) gene expression was not changed in all cell samples (Fig. 4I). A clear reduction of talin-1 protein was measured in MCS (Fig. 4J).

Integrin-β_1_ is a crucial cell surface receptor that mediates binding of the cell with its surrounding ECM. Additionally, it has a signaling function leading to modulation of gene expression within the cell^65^, and plays an important role in processing mechanotransduction^66^. Talin, together with fibronectin, laminin, integrin-β_1_ and vinculin, plays a major role in focal adhesion processes^12^. The same role applies to focal adhesion kinase (FAK/protein tyrosine kinase 2 (PTK2)) and E-cadherin, of which the latter is largely contributing to the formation of MCS^67^. In addition, FAK had been shown to play a crucial role in mechanosensing and -transduction^68^. It is also involved in migration, it links to the ECM and the cytoskeleton. After a three-day RPM-exposure, the *FAK1*/*PTK2* gene expression was not changed in all groups (Fig. 4K). FAs are composed of different functional modules separately controling mechanosensing and the cellular mechanoresponse. PTK2 (FAK) has shown to be involved in directing the cellular answer by controlling lamellipodial protrusions and cell migration^69^. FAK is involved in the regulation of adhesion, a process which occurs early. The unaltered gene expression after three days may be explained that the biological response of the cells to gravitational unloading occurred within minutes after starting the RPM and the exposure of the NHDF to µ*g*-conditions.

Moreover, we measured E-cadherin protein which is involved in cell-cell adhesion and necessary for wound healing^70^. E-cadherin protein was significantly enhanced in the MCS group vs. 1*g* and AD cells (Fig. 4L), which is an interesting finding and hints to its importance for 3D growth and MCS formation of fibroblasts. In contrast, in breast cancer cells exposed to r-µ*g* and s-µ*g* it was demonstrated that E-cadherin protein was significantly reduced and is involved in cell adhesion processes, and plays a significant role in tumorigenesis^11,71^. In addition, blockage of E-cadherin and a down-regulation of *CDH1* led to enhanced spheroid formation of MCF-7 breast cancer cells^11^. Changes in the E-cadherin protein synthesis can lead to tumor progression. Pathway analyses indicate that VCL protein has an activating effect on *CDH1*^71^.

Another important group of proteins known to be involved in 3D growth of cells are the caveolins. Caveolin-1 is involved in the induction of apoptosis and inhibits the TGF-β_1_-induced production of ECM. In contrast to previous data with thyroid cancer cells^13,72^, caveolin-1 (*CAV1*) was not changed in AD and MCS cells (Fig. 4M). However, the caveolin-1 protein content in AD was counter-regulated and significantly elevated compared to 1*g* and MCS (Fig. 4N). This finding is different as compared to data obtained from cancer cells, where caveolin has demonstrated to be a key inhibiting regulator of 3D growth in malignant thyroid cancer cells^72^. Obviously caveolin-1 has different effects in normal cells, which has to be further examined in the future. CAV1 has been associated with the regulation of cell mechanics, including cell softening and loss of stiffness sensing ability in NIH3T3 fibroblasts^73^. CAV1 controls the hyperresponsiveness of fibroblasts to mechanical stimulation^73^ and is involved in the progression of keloids during wound healing. The authors showed that an epigenetically decreased CAV1 elevated fibrogenesis-associated RUNX2 and influences the cell mechanics. Long-term studies of NHDF and NIH3T3 exposed to s-µ*g* and to vibrations using a Vibraplex device should be performed in the future.

### Growth factors, cytokines and collagen metabolism

In a next step, we investigated growth factors known to be involved in growth and proliferation. CTGF, EGF, transforming growth factor beta (TGF-β) and VEGF-A all contribute to the stimulation of growth in different cell types. *CTGF* and *EGF* mRNAs were up-regulated in thyroid cancer cells exposed to r-µ*g* in space and are responsible among others for the formation of MCS^16^.

**Figure 5:**
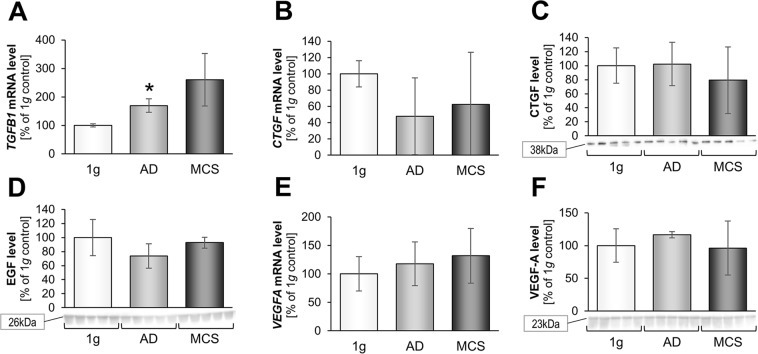
Investigation of selected growth factors after a 3-day s-µ*g*-exposure of normal human dermal fibroblasts: Quantitative gene expression levels of *TGFB1* (**A**). Transcriptional and translational connective tissue growth factor (CTGF) analysis: Quantitative gene expression levels of *CTGF* (**B**) and intracellular CTGF protein levels (**C**). Intracellular EGF protein levels (**D**). Transcriptional and translational vascular endothelial growth factor A (VEGF-A) analysis: Quantitative gene expression levels of *VEGFA* (**E**) and intracellular VEGF-A protein levels (**F**). Full-length blots of cropped Western blot images are presented in Supplementary Fig. S4. *p < 0.05 1*g* vs. RPM; ^#^p < 0.05 AD vs. MCS.

We detected a significant up-regulation of *TGFB1* gene expression of AD and a slight, not significant elevation in MCS as compared to 1*g*-control cells (Fig. 5A). TGF-β_1_ is a multifunctional cytokine and plays a major contextual role in cellular proliferation, adhesion and differentiation, consequently being a target in therapy of malignant diseases^74^. It is also stimulating adhesion, migration and influencing the growth behavior of normal cells.

*CTGF* gene expression and protein content were not significantly altered in AD and MCS cells (Fig. 5B,C). This is different compared to the results obtained from malignant cells like FTC-133 thyroid cancer cells. The *CTGF* gene expression was down-regulated in MCS of thyroid cancer cells exposed for three days to the 2D clinostat and RPM, whereas in the AD group the *CTGF* mRNA was not changed compared with 1*g*-controls^13^. In addition, we found no change of the *EGF* mRNA in AD and MCS cells (Fig. 5D). The reason for this different behavior of the fibroblasts may be their mesenchymal origin. It may be speculated that CTGF and EGF are not involved in 3D formation of normal fibroblast spheroids. This contrasts findings obtained from the Sino-German Shenzhou-8 spaceflight mission, where the experimental findings revealed a scaffold-free formation of extraordinary large 3D MCS by FTC-133 cells with an increased expression of *EGF* and *CTGF* genes under r-µ*g* and on the RPM^16^. These differences might be explained by the longer experiment duration of 10 days. The impact of launch stress and the influence of cosmic radiation may not play a role because the accompanying RPM experiment revealed a comparable result^16^.

In a further step, we focused on VEGF-A which plays a major role in cell growth and angiogenesis. Angiogenesis, the development of new vessels from existing vasculature, is important for tumor and normal cell growth, survival, and progression. On the molecular level, these biological processes are affected by a number of cytokines, among which the VEGF family and their related receptors, have an exceptional position^75^. A recent study showed a down-regulation of *VEGFA* in endothelial cells exposed to short-term r-µ*g* obtained during parabolic flight maneuvers and vibration^75^. VEGF-A has recently been described as an important regulatory protein in tube formation^12,76^. Our research group was able to show an elevated VEGF-A level in cellular supernatants of endothelial cells after a 35-day culture on the RPM^41^. In this study, the *VEGFA* gene expression and corresponding protein were not changed after a 3-day RPM-culture (Fig. 5E,F).

Moreover, we investigated the synthesis and release of gravi-sensitive cytokines (IL-6, IL-8, MCP-1) and the expression of *TNFA, RELA, ICAM1* and *JNK1*. Recently, we demonstrated the profound influence of interleukin-6 (IL-6) and interleukin-8 (IL-8) on spheroid formation in cancer cells, when cultured on the RPM and under 1*g*-conditions, applying the liquid-overlay technique^23^. Hence, we focused - among others - on these interleukins to further characterize the signaling pathways in NHDF. Additionally, an investigation of the cytokine levels in the supernatants of NHDF cells was performed using Multi-Analyte Profiling (MAP) technology (Human Cytokine MAP^®^ A v1.0, Myriad RBM, Inc.). Of all investigated cytokines (GM-CSF, IFN-γ, IL-2, IL-3, IL-4, IL-5, IL-6, IL-7, IL-8, IL-10, IL-18, MIP-1α, MIP-1β, TNFα, TNF-β) only three (IL-6, IL-8, MCP-1) showed a substantial occurrence above the lower limit of quantification (LLOQ). The soluble proteins identified by MAP were measured regarding their mutual interactions and their influence on proteins, which were associated with the cells secreting the soluble proteins and had been identified in earlier studies^12,13,77^.

**Figure 6:**
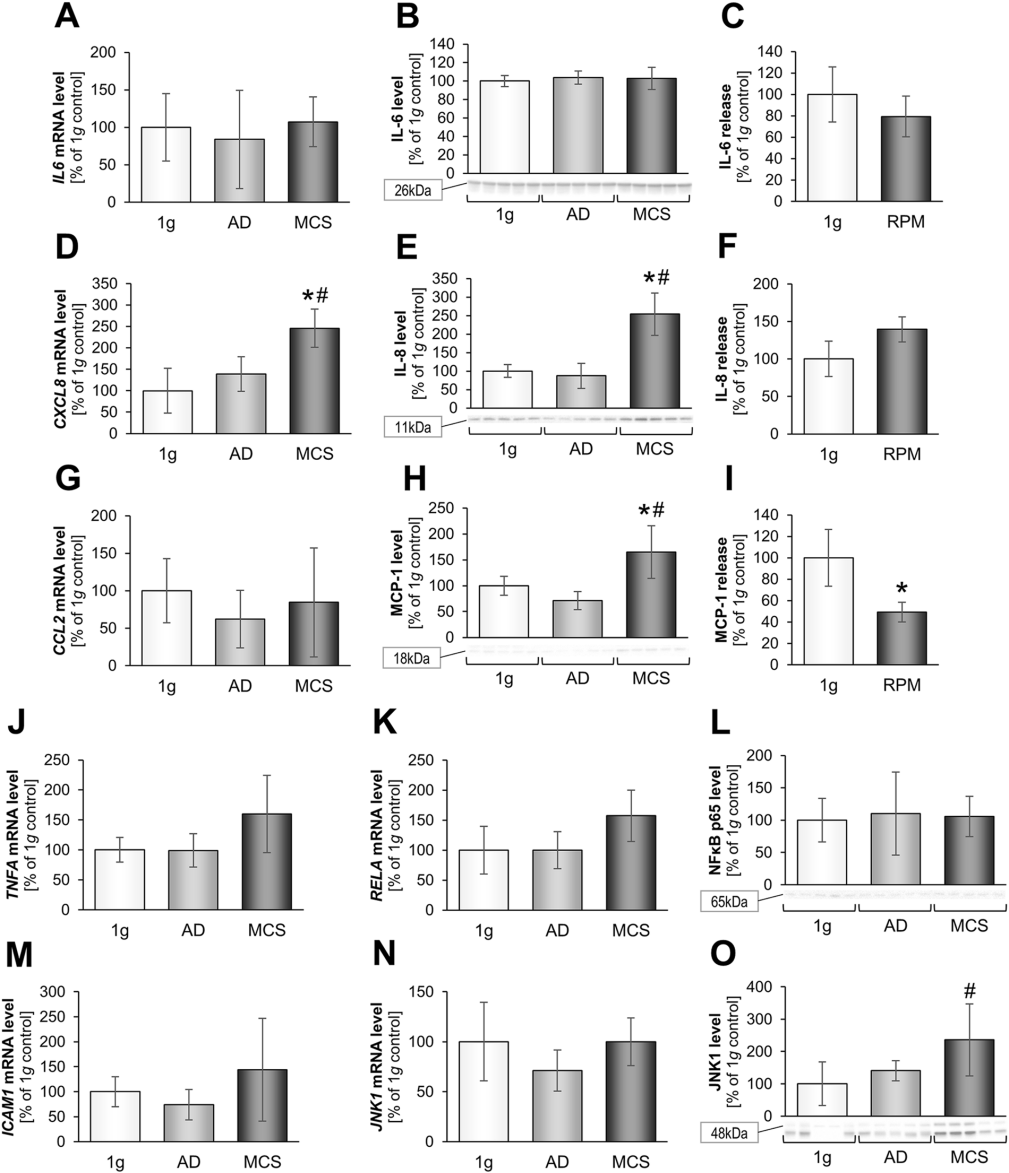
Analysis of selected cytokines after a 3-day s-µ*g*-exposure: Transcriptional and translational IL-6 analysis: Quantitative gene expression levels of *IL-6* (**A**) and intracellular IL-6 levels (**B**), release of IL-6 (**C**). Transcriptional and translational IL-8 analysis: Quantitative gene expression levels of *CXCL8* (**D**) and intracellular IL-8 levels (**E**), release of IL-8 (**F**). Transcriptional and translational MCP-1 analysis: Quantitative gene expression levels of *CCL2* (**G**), intracellular MCP-1 levels (**H**), release of MCP-1 (**I**). Quantitative gene expression levels of *TNFA* (**J**). Transcriptional and translational NF-κB p65 analysis: Quantitative gene expression levels of *RELA* (**K**) and intracellular NF-*κ*B p65 levels (**L**). Quantitative gene expression levels of *ICAM1* (**M**). Transcriptional and translational JNK1 analysis: Quantitative gene expression levels of *JNK1* (**N**) and intracellular JNK1 protein levels (**O**). Full-length blots of cropped Western blot images are presented in Supplementary Fig. S5. *p < 0.05 1*g* vs. RPM; ^#^p < 0.05 AD vs. MCS.

The *IL6* gene expression and protein content were not changed (Fig. 6A,B). The IL-6 protein secretion was slightly reduced in RPM-samples (Fig. 6C). In contrast, we measured a significant increase in the *IL8* (*CXCL8*) gene expression (Fig. 6D) and the amount of IL-8 protein in MCS (Fig. 6E). This was paralleled by a tendentially higher secretion of IL-8 protein in the RPM group (Fig. 6F). However, the changes in IL-6 and IL-8 correspond with our findings from previous studies^12,23,41^. Interestingly, the obtained MAP analysis is coherent with the respective Western blot data. The interleukin IL-8 had been suggested to induce the expression of several proteins like β-actin, integrin-β_1_ and talin, and support their key role in tumor progression, metastasis and angiogenesis^23,78^. Grosse *et al*. showed in 2012 that IL-6, IL-8, OPN, TLN1, CTGF, and NF-κB play a large contributory role in RPM-dependent thyroid carcinoma cell spheroid formation^79^. A direct influence of IL-8 protein on MCS engineered under normal 1*g-*conditions using the liquid-overlay technique was demonstrated on FTC-133 and ML-1 follicular thyroid cancer cell cultures^23^.

In addition, we focused on monocyte chemoattractant protein-1 (MCP-1, C-C motif chemokine ligand 2 (CCL2)), which is a key member of the large family of chemokines regulating mainly cell trafficking. MCP-1 itself is associated with regulating migration and infiltration of monocytes/macrophages^80^. A large number of studies focused on MCP-1 and its involvement in various diseases. A high or constitutive expression level of CCL2 is often observed^81^. The human thyroid Nthy-ori 3-1 cells secrete MCP-1 after 24 h in a continually increasing amount, although no differences could be observed between normal gravity and s-μ*g*^12^. The gene expression of *CCL2* was not changed (Fig. 6G), but MCP-1 protein was significantly increased in MCS cells (Fig. 6H). Furthermore, the MAP analysis showed a clearly decreased MCP-1 level in RPM supernatants (Fig. 6I). An up-regulation of the *CCL2* mRNA was also measured in AD cells and a down-regulation of MCP1 was also found in UCLA RO-82-W-1 follicular thyroid cancer spheroids formed after a 24-hour RPM-exposure^77^.

In addition, we investigated tumor necrosis factor alpha (TNFα) (Fig. 6J) and nuclear factor ‘kappa-light-chain-enhancer’ of activated B-cells (NF-κB) (Fig. 6K,L), which are both playing a role in apoptosis, cell survival and cell detachment^79^. *TNFA* was not altered in RPM samples, which indicate that it is not supporting the MCS formation of NHDF (Fig. 6J). It was earlier reported that FTC-133 thyroid cancer cells grown on the RPM showed higher levels of NF-κB p65 (RelA) protein and apoptosis than 1*g*-controls, a result also found earlier in endothelial cells^79^. The V-rel avian reticuloendotheliosis viral oncogene homolog A (*RELA*) mRNA remained unchanged in all groups (Fig. 6K). Furthermore, NF-κB p65 protein was not significantly altered (Fig. 6L). Therefore, NF-κB p65 seems not to be involved in the RPM-dependent NHDF spheroid formation, which is in contrast to tumor cells. In earlier studies, we had demonstrated the involvement of NF-κB p65 in MCS formation of thyroid cancer cells^79^ and MCF-7 breast cancer cells^8^. To our knowledge this is the first report that NF-κB p65 is not influencing the 3D growth of NHDF.

Finally, we evaluated the *ICAM1* gene expression in this setting and could demonstrate that *ICAM1* in NHDF was not significantly altered by RPM-exposure (Fig. 6M). Other investigated cytokines were c-Jun N-terminal kinases (JNKs), known to be involved in apoptosis, neurodegeneration, cell differentiation and proliferation^31^.

JNK1 or mitogen-activated protein kinase 8 is a mediator to cellular external stimuli that plays a contributory role in TNFα-mediated apoptosis through MAPK/JNK pathway^82^ and ICAM-1, a surface protein contributing to cell-cell interaction and interacting with integrin-β_1_ and believed to promote MCS formation^8^. However, JNK1 levels in MCS were not significantly changed compared to 1*g*, but significantly elevated compared to AD (Fig. 6N,O).

To our best knowledge this is the first report showing that IL-8 might be an important trigger of spheroid formation in dermal fibroblasts, while IL-6 and NF-κB p65 which are both important inducers of metastasis in cancer seem not to be involved in 3D growth in benign fibroblasts. Future functional tests will prove these observations.

Moreover, we studied the collagen synthesis and metabolism of NHDF and focused on collagen type I, III and IV, as well as on the expression of *TIMP1, MMP1, MMP3*, and *MMP14*. As previously mentioned, fibroblasts are the key cell type to maintain a proper dermal function. They synthesize collagen, the main structural component of the skin providing a structural framework and elasticity. Lack of collagen or improper collagen homeostasis (e.g. in the aging skin or after intensive UV-radiation photodamage) leads to a deficient dermal structure^83,84^. In general, the collagen homeostasis is maintained via collagen synthesis by fibroblasts and the modulation by matrix metalloproteinases (MMPs). These MMPs themselves are regulated by tissue inhibitors of metalloproteinases (TIMPs)^85^. Photoaged skin (e.g. through UV-radiation) is often characterized by a distinct damage of dermal connective tissue and a malfunction of the collagen-MMP-TIMP regulation^86,87^. MMP1 (interstitial collagenase) and MMP3 (stromelysin-1) have been shown to be elevated with UV-radiation damage to skin^84^. MMP1 levels have also been elevated in aged skin or been correlated with collagen fragmentation that resembles a malfunctioning dermal ECM microenvironment^88,89^.

As astronauts in space encounter a variety of these environmental factors like UV radiation and vibration, it is of interest to investigate the fibroblast reaction, particularly with regard to s-µ*g*, where this interplay of environmental factors is reduced to only one.

**Figure 7:**
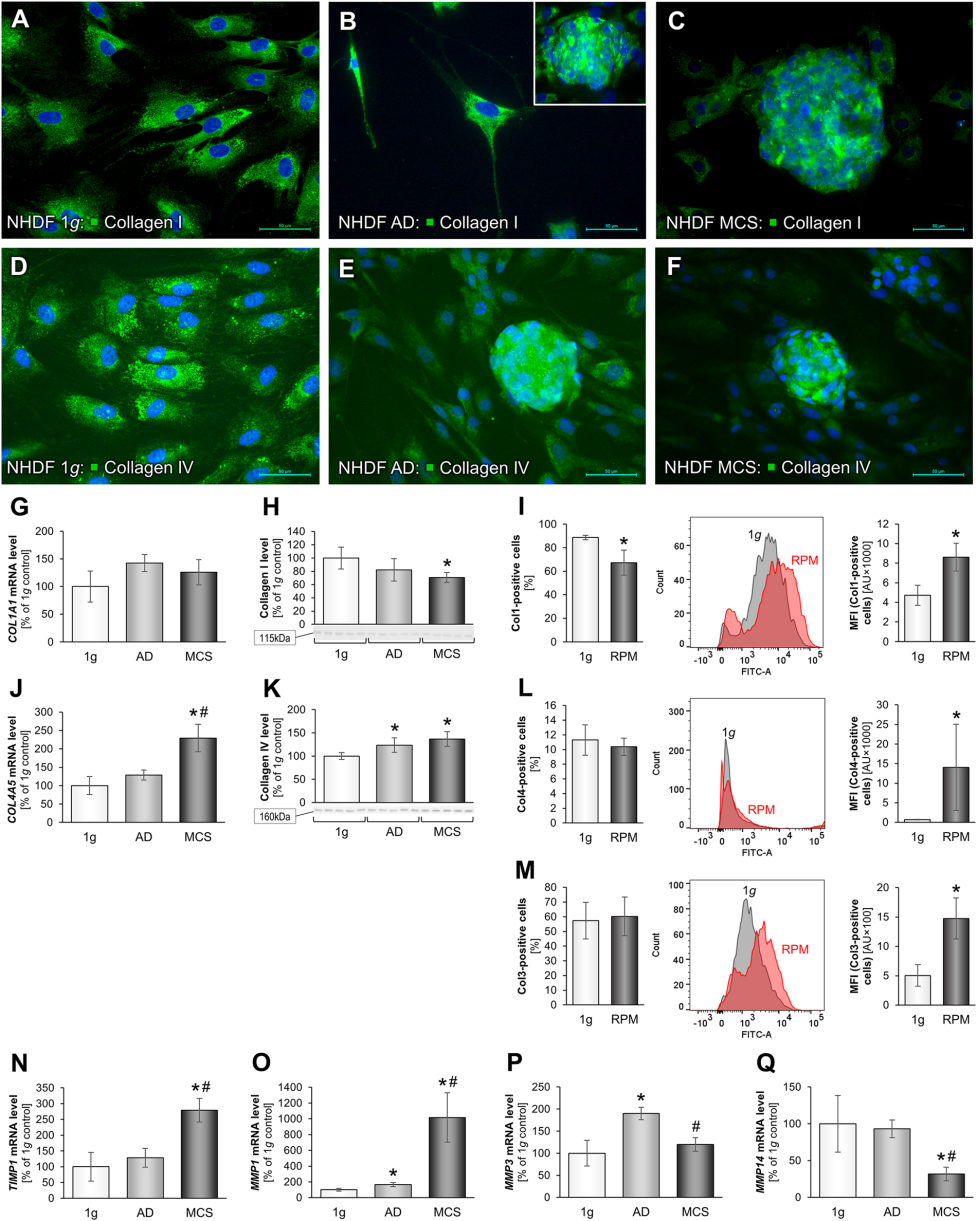
Alterations in collagen levels of juvenile NDHF cells following a 3-day exposure to microgravity: Immunofluorescence images of 1*g*-control cells, RPM-AD and RPM-MCS of collagen-I (**A**–**C**) and collagen-IV (**D**–**F**). Transcriptional and translational collagen type I analysis: Quantitative gene expression levels of *COL1A1* (**G**), intracellular collagen-I levels (**H**) and flow cytometric analysis of collagen type I-labeled cells (**I**). Quantitative gene expression levels of *COL4A5* (**J**), intracellular collagen-I levels (**K**) and flow cytometric analysis of fibronectin-labeled cells (**L**). Flow cytometric analysis of collagen type III-labeled cells (**M**). Quantitative gene expression levels of *TIMP1* (**N**), *MMP1* (**O**), *MMP3* (**P**) and *MMP14* (**Q**). Full-length blots of cropped Western blot images are presented in Supplementary Fig. S6. *p < 0.05 1*g* vs. RPM; ^#^p < 0.05 AD vs. MCS. Scale bars: 50 µm.

IFS of collagen types I and IV revealed a relatively equal distribution within the three groups (Fig. 7A–F). The *COL1A1* gene expression (Fig. 7G) was not changed in all RPM samples, but the corresponding protein was reduced in MCS samples as measured by Western blot (Fig. 7H). In addition, the flow cytometry analyses showed a reduced amount of collagen type I-positive AD cells, but with a higher MFI (Fig. 7I).

Regarding the gene expression profile, the obtained data showed a highly significant up-regulation of *COL4A5* in MCS samples (Fig. 7J). This was confirmed by Western blot analysis (Fig. 7K). Flow cytometry revealed no change in the amount of collagen type IV-positive cells, but an increase in MFI (Fig. 7L). Collagen type IV is an essential contributory part to the cellular basal laminae, which it forms together with entactin, perlecan and laminin^90,91^. Comparing these results with those of laminin protein, this data hints towards a distinct influence on the cellular basal lamina configuration, when cells are exposed to s-µ*g*. In addition, we examined the cells concerning collagen type III positivity by flow cytometry and measured no changes in 1*g* and RPM samples, but the RPM cells showed an elevated MFI (Fig. 7M). Collagen types I and III are structural proteins of the dermal ECM^92,93^. As collagen type I is synthesized in its pro-collagen precursor state by fibroblasts it forms fibrils that are stabilized by cross-linkage^84^.

Interestingly, in this study, there was also a significant up-regulation of the *TIMP1* and *MMP1* gene expression in MCS compared with the 1*g*-control group (Fig. 7N,O). In contrast, MMP1 and *MMP3* were both significantly up-regulated in AD samples. Tissue inhibitor of metalloproteinases inhibit metalloproteinases, which in turn are widely known to degrade the ECM and thus participate in remodeling the shape and composition of cell aggregates and tissues^12^. An overexpression of *TIMP1* was also implicated in several cancer types and correlated with a less-positive outcome for a patient after treatment^94^. An increase in TIMP1 might be a facilitator of cell-cell contacts during the rearrangement of the focal adhesion complex for 3D cell aggregate formation^12,95^. The matrix metalloproteinase 1 (MMP1) is also known as fibroblast collagenase, which is responsible for tissue remodeling and the breakdown of collagen type I, II and III. We also examined MMP3 which breaks down various collagen types (II, III, IV, IX), and other ECM proteins like proteoglycans, fibronectin, or laminin. In addition, MMP3 can activate MMP1, MMP7 or MMP9 revealing that MMP3 is essential in tissue remodeling. The *MMP3* gene was up-regulated in AD cells compared to 1*g* (Fig. 7P).

Finally, we focused on MMP14, which is important for the structural remodeling during aging processes. MMP14 expression in fibroblasts is important for the collagen remodeling in adult skin and supports the dermal homeostasis^96^. *MMP14* was clearly decreased in MCS compared to 1*g*-controls (Fig. 7Q).

Taken together, the expression and amount of the basal lamina component collagen type IV was significantly elevated in MCS, indicating its importance for the 3D growth of fibroblasts. In parallel, *TIMP1*, *MMP1* mRNAs were significantly up-regulated and *MMP14* down-regulated in MCS indicating their involvement in 3D growth. The meaning of these findings has to be studied in more detail in the future.

### *In silico* analysis of relationships and interaction of studied genes and proteins

**Figure 8:**
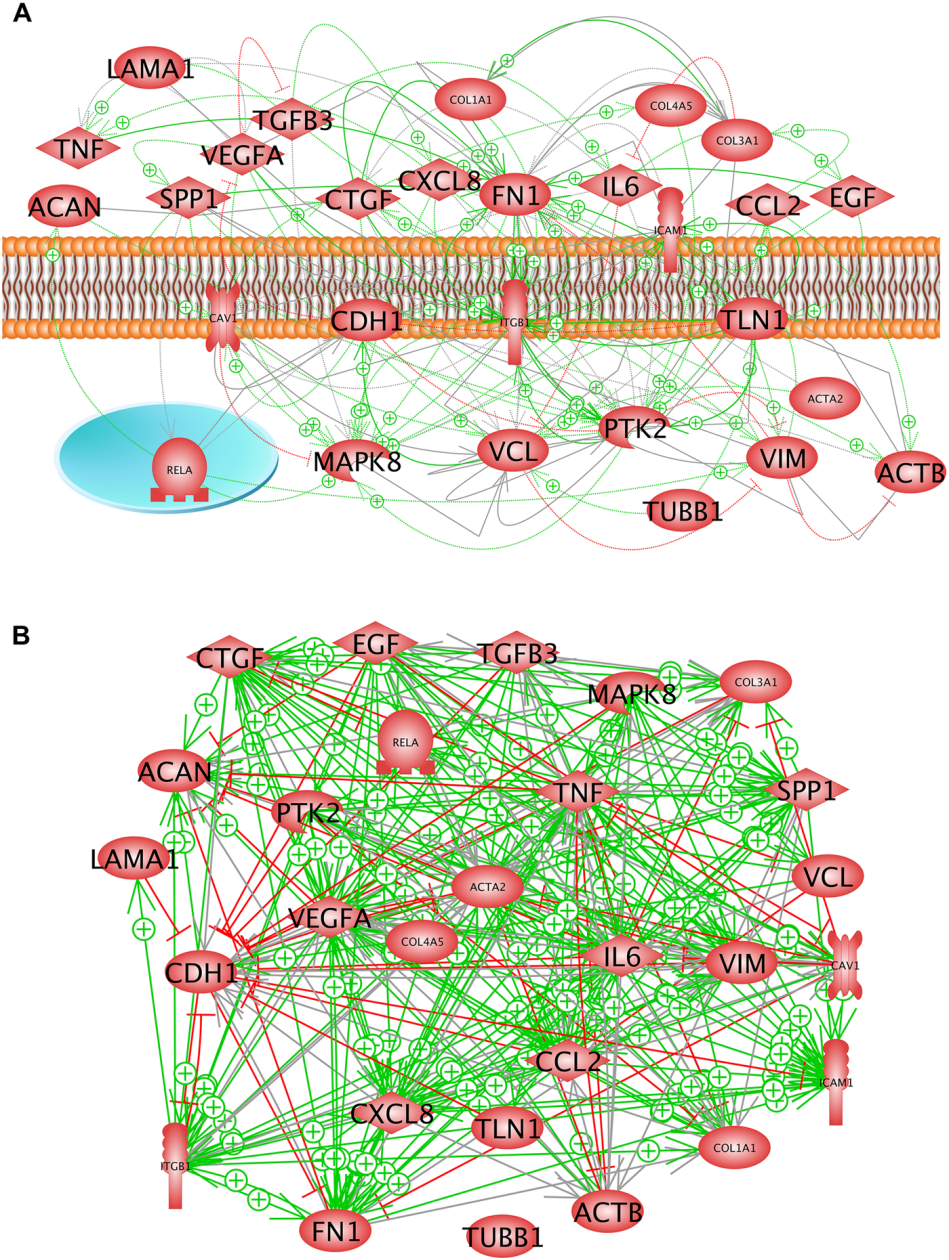
(**A**) Pathway studio analysis of investigated proteins. The figure shows all occurring protein-protein interactions. The color of shown lines indicates the type of interaction: green line: promotion of indicated partner; red line: inhibition of indicated partner. Grey solid lines indicate possible complex formation, solid arrows point to direct interaction and dashed arrows to indirect influence. Membrane and nucleus (blue circle) mark predominant localization of the proteins. (**B**) Pathway studio analysis on the expression of investigated genes. The color of shown lines indicates the type of interaction: green line: promotion of indicated partner; red line: inhibition of indicated partner. Arrows indicate the direction of influence.

After a cohort of 27 proteins and genes coding for them has been investigated regarding their accumulation and regulation, respectively, we focused on the proteins’ cellular localization and their mutual relationships. The *in silico* analysis of the proteins mentioned above indicated 15 proteins, which are mainly active in the extracellular space, five proteins normally inserted in the membrane and seven proteins of the cytoplasm or the nucleus. They form an extended network of 48 direct interactions shown by solid lines or arrows and of another 60 ways of mutual influence as indicated by dashed arrows (Fig. 8A). In this system fibronectin and integrin-β_1_ play a central role. When activated and forming a heterodimer with an appropriate integrin-alpha, integrin-β_1_ can bind to fibronectin, laminin, osteopontin and various collagens via the extracellular domain^97,98^. Integrin-β_1_ is activated by binding talin and kindlin via its cytoplasmic domain^99^. The signal of this binding is transferred to the cytoskeleton and the FA complex via vinculin and actin^100^. Vinculin binds to mitogen-activated protein kinase 8 (MAPK8)^101^ and mechanosensing of E-cadherin^102^. Both components have an influence on NF-κB (RELA, Fig. 8A). Caveolin-1 regulates integrin-β_1_ and SRC by forming complexes with both components of the focal FA system^103^. Production of fibronectin is stimulated by the epidermal growth factor^104^. EGF was slightly reduced in fibroblasts exposed to the RPM (Fig. 5D). This could, according to the presented analysis, be a reason that E-cadherin is enhanced and integrin-β_1_ remains unchanged. Figure 8B shows the influence of the proteins on the expression of the relevant genes. This network includes 244 relationships. Looking at the green arrows shown it can be seen that most of the arrows start at MAPK8 or EGF. This means that both are main up-regulators of the system. While, as indicated above, the EGF protein was slightly lowered in AD cells during incubation on the RPM (Fig. 5D), the expression of *MAPK8* (*JNK1*) remained stable under s-µ*g* compared to 1*g* (Fig. 6N,O). Both entities have equal and different targets, as shown by the arrowheads. A possible balance of both systems appears to be a topic of future research.

## Conclusions

Juvenile NHDF, when exposed to s-µ*g*, grew in form of two different phenotypes, as adherent monolayer and as cells in compact MCS (Fig. 1). A similar behavior is also shown by other cell types, when they are cultured in space or under s-µ*g* conditions on Earth. Benign cells (chondrocytes^105^ or thyroid cells^12^) and thyroid cancer cells^13,106^ also exhibited a comparable growth. As mentioned above and shown by the various flow cytometric measurements, the reason for this phenomenon is probably due to a different accumulation of distinct, for spheroid formation responsible proteins in the individual cells of a population. The differences between the individual cells appear to be more expressed in cell populations cultured on the RPM as compared to the 1*g*-cultures (Figs 2 and 7). Though, at the moment one can only speculate about that spreading of protein accumulation. It could be due to differences of localization of the cells in a culture flask or to a variation of protein production based on regulation of transcription and translation processes. Perhaps both factors play a role.

The differential regulation of several ECM proteins, growth factors, cytokines in connection with matrix metalloproteinases showed a picture of interacting proteins (positively or negatively) which clearly strengthens 3D growth of the cells exposed to s-µ*g*. The differences and similarities between the adherent population and MCS are summarized in Table 1. Major divergences occurring among these two clusters are measured for vimentin, laminin, collagen type IV, TIMP1, MMP1, MMP14, E-cadherin, and IL-8. A similar result of both phenotypes was found for TGF-β_1_, ITGB1, FN1, SPP1, and CAV1. These data suggest to evaluate the functional effects of these proteins in future investigations. In addition, future studies will be performed to shed light on the mechanisms involved in the process of spheroid formation, such as genetic knock-outs or knock-ins in cell lines. The membrane components laminin and collagen type IV, fibronectin, IL-8, MMPs and TIMP1 in concert with TGF-β_1_ and integrin-β_1_ play a major role in our experimental setting.

**Table 1:** Biochemical differences between adherently growing (2D) and multicellular spheroid cells (3D growth) of NHDF in s-µ*g*.

Structure	AD cells	MCS cells
**Focal adhesions**		
gene expression	*ITGB1*↑	
intracellular protein	Caveolin-1↑	E-cadherin↑, Talin-1↑
**Extracellular matrix**		
gene expression	*FN1*↑ *MMP1*↑, *MMP3*↑	*LAMA3↑*, *ACAN*↓, *COL4A5*↑, *TIMP1*↑, *MMP1*↑↑, *MMP14*↓
intracellular protein	Fibronectin↑ Osteopontin↑ Collagen IV↑	Fibronectin↑ Osteopontin↑↑ Aggrecan↓ Collagen I↓, Collagen IV↑
**Cytoskeleton**		
intracellular protein	Vimentin↑	Pan-Actin*↑*, α-SM Actin↓
**Growth factors**		
gene expression	*TGFB1*↑	
**Cytokines**		
gene expression		*CXCL8*↑
intracellular protein		IL-8↑, MCP-1↑, JNK1↑
protein release		MCP-1↓

These new findings with NHDF indicate what may possibly occur in space. The behavior of fibroblasts under space conditions is of high interest because the astronaut’s skin is getting thinner (thinned stratum corneum, impaired barrier function, and loss of dermal elasticity)^3,107^ with time. Research in this area is of high interest because of the planned space tourism adventures in the near future. Moreover, knowledge about the mechanisms of fibroblast spheroid formation helps to increase our understanding in the biofabrication of biological tissues or it can be used for co-culture experiments with cancer cells and following drug testing.

## Material and Methods

### Cell culture

Juvenile normal human dermal fibroblasts (NHDF, catalogue number C-12300; PromoCell GmbH, Heidelberg, Germany) were delivered in a cryo-conserved state and cultured in 75 cm^2^ ventilated cell culture flasks (Sarstedt AG, Nürnbrecht, Germany). Roswell Park Memorial Institute Medium (RPMI 1640) (Gibco, Carlsbad, USA) supplemented with 10% fetal calf serum (FCS) (ThermoFisher, Waltham, USA), 100 IU penicillin and 100 µg streptomycin (Biochrom AG, Berlin, Germany) was used under standard conditions of 37 °C and 5% CO_2_. The RPM experiments including control groups were performed by seeding juvenile fibroblasts in 25 cm^2^ ventilated cell culture flasks (Sarstedt AG) and allowing 24 h for the cells to attach to the bottom of the flask. A day later, the experiment started. In order to reduce shear stress from air inclusions, flasks were filled completely with medium. After completion of experiments medium was centrifugated thoroughly for detection of multicellular spheroids (MCS).

### Random positioning machine

**Figure 9:**
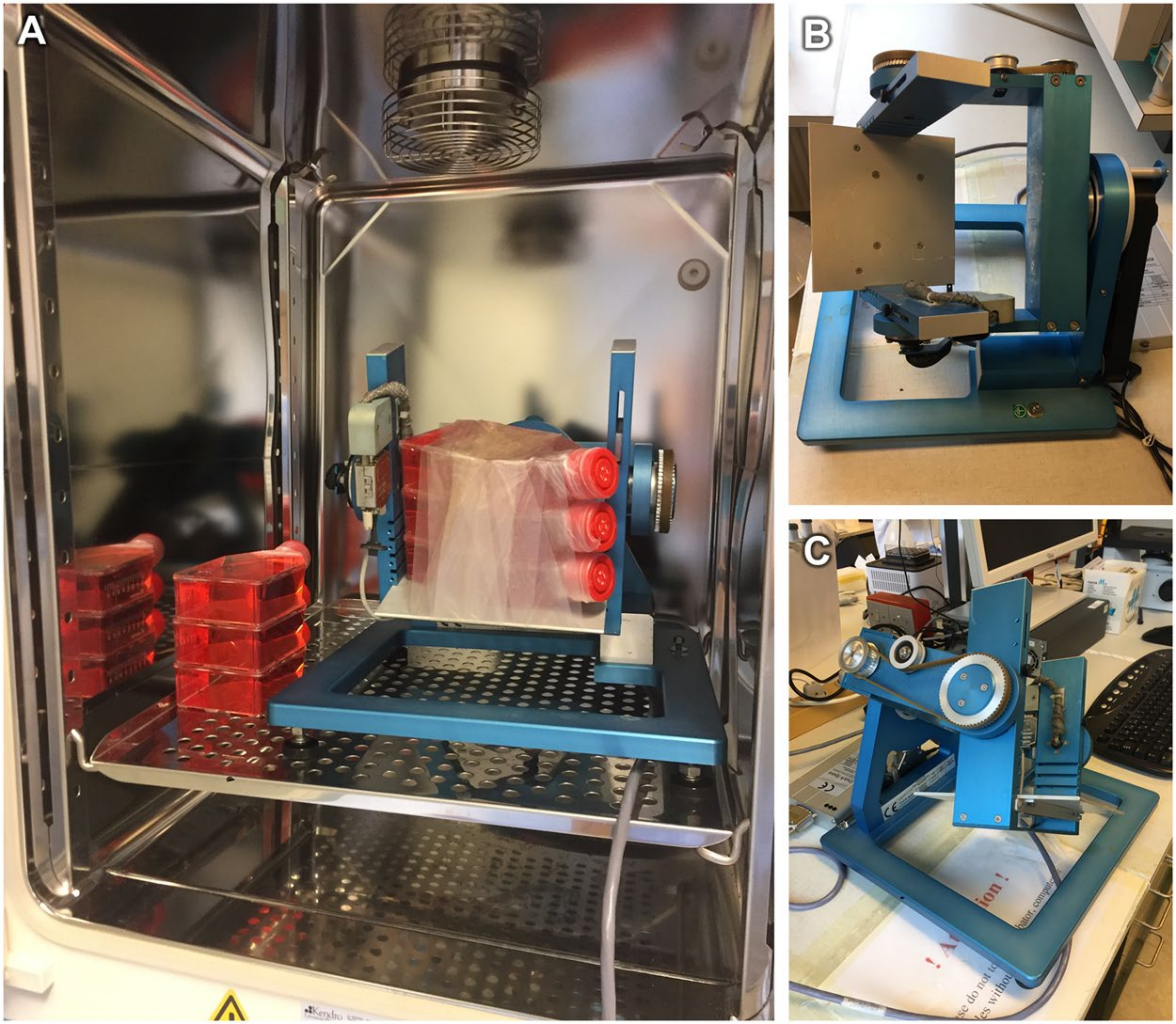
Table-top random positioning machine: Mounted cell culture flasks for RPM-experiments with adjacent 1*g-*control flasks in an incubator (**A**). RPM-frame without mounted flasks (**B**,**C**).

All experiments for simulation of the microgravity conditions were performed using a desktop RPM (Airbus Defence and Space, former Dutch Space, Leiden, Netherlands). The RPM was described earlier in detail^13,108,109^. Briefly, the RPM consists of two perpendicular, independently motor-driven frames, with the innermost frame carrying the specimen to be exposed to s-µ*g*. Using a personal computer, the frames were operated in the 3D random mode, moving in random directions and speeds, maintaining a mean angular velocity of 60 °/s. Over time, this movement pattern will lead to an averaging of the gravitational vector to zero, effectively producing a state of s-µ*g*. The quality of the achieved s-µ*g* is best in the center of the two rotational axes while some residual acceleration forces are present outside of it. Cell cultures of the RPM-groups were seeded as described above and mounted to the RPM platform. The RPM was placed inside an incubator at 37 °C and 5% CO_2_^13^. Cell cultures of the 1*g*-control groups were placed next to the RPM inside the same incubator (Fig. 9A).

### Microscopy

Phase contrast microscopy was performed before and after the RPM-experiments to ensure cell viability and to detect possible morphological changes of the cell cultures. Phase contrast pictures were taken with a Leica DM IL LED microscope equipped with a Leica DFC310 FX digital CCD color camera (Leica Microsystems, Wetzlar, Germany). Immunofluorescence-stained cells were analyzed with a Leica DM2000 microscope with light source Leica EL6000. Excitation and emission wavelengths were λ_exc_ = 488 nm and λ_em_ = 505 nm for FITC. All samples were analyzed with the help of the image analysis program Leica Application Suite (Version 3.7; Leica Microsystems, Wetzlar, Germany).

### Hematoxylin-Eosin and Elastica van Gieson staining

After the 3-day RPM-culture of NHDF, the 1*g*-cells, adherent s-µ*g*-cells and MCS were carefully washed three times in phosphate buffered saline (PBS) and fixed in 4% paraformaldehyde. HE- and EvG-staining procedures were performed to evaluate the cell morphology of the NHDF as previously described^53,110,111^. Briefly, for the HE stain, the cells were fixed with 2% PFA (Carl Roth) for 10 min. Following the fixation, the cells were incubated for 5 min with distilled water, then incubated for 10 min with an acidic Mayer’s hematoxylin solution (Carl Roth) to stain the cell nuclei blue. The dye was removed by washing the sample three times with tapwater.The cytoplasm and other cell structures were stained with eosin G (Carl Roth) for 10 min and then washed three times with tapwater. The EvG staining was done according to standard procedures by the routine diagnostic laboratory of the Institute of Pathology, University Hospital of Regensburg. All samples were visualized by light microscopy, using the Leica DM IL LED microscope, as described above.

### Immunofluorescence staining

The cells were fixed with a 4% paraformaldehyde solution for 20 min. Then they were subjected to membrane permeabilization with ice-cold methanol for 10 min and blocking with 1% (weight (w)/volume (v)) bovine serum albumin (BSA, Sigma-Aldrich, Steinheim, Germany) in PBS for 15 min. The slides were then released from the flasks and the cells were incubated with primary antibodies (listed in Table 2) in a PBS solution with 1% (w/v) BSA for 24 h at room temperature, followed by washing and incubation with secondary antibodies (Table 2) in PBS solution with 1% (w/v) BSA for an additional 24 h. Afterwards, the cells were washed three times with PBS and prepared for microscopy using Fluoroshield™ mounting media with DAPI (4’,6-diamidino-2-phenylindole; Sigma-Aldrich). Slides were kept at 4 °C in a dark box for 2 days before microscopical investigation^41^.

**Table 2:** Antibodies used for immunofluorescence staining (IFS).

Primary Antibody	Company	Dilution	Molecular Weight (kDa)	Catalogue Number
Collagen I	Invitrogen	1:250	115–120	PA1-26204
Collagen IV	Invitrogen	1:250	160	PA5-50939
Fibronectin	Sigma	1:400	220	F3648
Laminin	Sigma	1:200	250	L9393
Vimentin	Sigma	1:200	53	V5255
Vinculin	Sigma	1:400	116	V9131

The corresponding secondary antibodies – Anti-rabbit IgG (H + L), F(ab’)_2_ Fragment (Alexa Fluor^®^488 Conjugate) (Ref. 4412S) and Anti-mouse IgG (H + L), F(ab’)_2_ Fragment (Alexa Fluor^®^488 Conjugate) (Ref. 4408S, both CST, Danvers, MA, USA) – were used at a dilution of 1:500.

### Flow cytometry

The staining procedure for flow cytometry was published earlier^14,112^. Briefly, PFA-fixed cells were carefully scraped off the bottom of the culture flasks, collected in 50-mL tubes and centrifuged for 5 min at 1500 × *g* at room temperature. The pelleted cells were resuspended in PBS, and 10^5^ cells each were transferred into two round-bottom tubes and incubated with 0.1% Triton X-100 in PBS for 10 min. The cells in one tube were incubated for 90 min with the respective primary antibody, while the other was incubated with PBS. The cells in both round-bottom tubes were then incubated with the secondary antibody (Cell Signaling; 1:1000) for 60 min. An overview of the used antibodies and their applied concentrations is given in Table 3. The final measurement of the single cells was performed with a FACSCanto™ II (Becton Dickinson, Franklin Lakes, NJ, USA). The analysis of the flow cytometry data was carried out with FlowJo 10.4 (FlowJo LLC, Ashland, OR, USA).

**Table 3:** Antibodies used for Flow Cytometry.

Primary Antibody	Company	Dilution	Molecular Weight (kDa)	Catalogue Number
Chondroitin sulfate	Invitrogen	1:100	210–250	MA3-16888
Collagen I	Invitrogen	1:100	115–120	PA1-26204
Collagen III	Invitrogen	1:100	140	PA5-27828
Collagen IV	Invitrogen	1:100	160	PA5-50939
Fibronectin	Invitrogen	1:100	220	PA5-29578
Laminin	Invitrogen	1:100	250	PA1-16730
Osteopontin	Sigma	1:100	50	SAB4200018
Vinculin	Abcam	1:100	116	Ab18058

The corresponding secondary antibodies – Anti-rabbit IgG (H + L), F(ab’)_2_ Fragment (Alexa Fluor^®^488 Conjugate) (Ref. 4412S) and Anti-mouse IgG (H + L), F(ab’)_2_ Fragment (Alexa Fluor^®^488 Conjugate) (Ref. 4408S, both CST) – were used at a dilution of 1:1000.

### RNA isolation and quantitative real-time PCR

The method of RNA extraction and qPCR was published earlier in detail^8,10,13^. Briefly, the RNA isolation was done using the RNeasy Mini Kit (Qiagen, Hilden, Germany), with an additional DNase digestion step (Qiagen) in order to eliminate residual DNA contaminations. Afterwards, the amount of RNA was quantified using a Photometer Ultrospec2010 (Amersham Biosciences, Freiburg, Germany). The first strand cDNA synthesis kit (Thermo Fisher Scientific, Waltham, US) was applied for reverse transcription. qPCR was performed on the 7500 Fast Real-Time PCR System using the FAST SYBR Green Master Mix (both Applied Biosystems, Darmstadt, Germany) according to standard protocols^8,10,13^. In short, 1 µg of isolated RNA was reverse-transcribed in a total volume of 20 µL reaction mix. For the PCR, 1 µL of the resulting cDNA was mixed with water, primer stocks and 2x FAST SYBR Green Master Mix to yield a final reaction voume of 15 µL with an end concentration of 300 nM for the primers. For the actual PCR run we employed the default protocol for the FAST Master Mixes as implemented in the system software, complemented by a subsequent melting curve analysis to check for primer specificity. cDNA-selective-primers were synthesized by TIB Molbiol (Berlin, Germany) and are listed in Table 4. The primers were designed using Primer Express (Applied Biosystems) to have a T_m_ of ∼60 °C and to span exon-exon boundaries. All samples were measured in triplicate. For normalization, 18S rRNA was used as a housekeeping gene. The comparative CT(ΔΔCT) method was used for relative quantification of transcription levels and 1*g* was defined as 100% for reference.

**Table 4:** Primer sequences for qPCR.

Gene	Primer Name	Sequence
*18S* (HKG)	18S-F	GGAGCCTGCGGCTTAATTT
	18S-R	CAACTAAGAACGGCCATGCA
*ACAN*	ACAN-F	AGTCCAACTCTTCAAGGTGAACTA
	ACAN-R	ACTCAGCGAGTTGTCATGGT
*ACTA2*	ACTA2-F	GAGCGTGGCTATTCCTTCGT
	ACTA2-R	TTCAAAGTCCAGAGCTACATAACACAGT
*ACTB*	ACTB-F	TGCCGACAGGATGCAGAAG
	ACTB-R	GCCGATCCACACGGAGTACT
*CAV1*	CAV1-F	CCTCCTCACAGTTTTCATCCA
	CAV1-R	TGTAGATGTTGCCCTGTTCC
*COL1A1*	COL1A1-F	ACGAAGACATCCCACCAATCAC
	COL1A1-R	CGTTGTCGCAGACGCAGAT
*COL4A5*	COL4A5-F	GGTACCTGTAACTACTATGCCAACTCCTA
	COL4A5-R	CGGCTAATTCGTGTCCTCAAG
*CTGF*	CTGF-F	ACAAGGGCCTCTTCTGTGACTT
	CTGF-R	GGTACACCGTACCACCGAAGAT
*FAK1*/*PTK2*	FAK1-F	TGTGGGTAAACCAGATCCTGC
	FAK1-R	CTGAAGCTTGACACCCTCGT
*FN1*	FN1-F	AGATCTACCTGTACACCTTGAATGACA
	FN1-R	CATGATACCAGCAAGGAATTGG
*ICAM1*	ICAM1-F	CGGCTGACGTGTGCAGTAAT
	ICAM1-R	CTTCTGAGACCTCTGGCTTCGT
*IL6*	IL6-F	CGGGAACGAAAGAGAAGCTCTA
	IL6-R	GAGCAGCCCCAGGGAGAA
*IL8*/*CXCL8*	IL8-F	TGGCAGCCTTCCTGATTTCT
	IL8-R	GGGTGGAAAGGTTTGGAGTATG
*ITGB1*	ITGB1-F	GAAAACAGCGCATATCTGGAAATT
	ITGB1-R	CAGCCAATCAGTGATCCACAA
*JNK1*/*MAPK8*	JNK1-F	TCTCCTTTAGGTGCAGCAGTG
	JNK1-R	CAGAGGCCAAAGTCGGATCT
*LAMA3*	LAMA3-F	AAAGCAAGAAGTCAGTCCAGC
	LAMA3-R	TCCCATGAAGACCATCTCGG
*MCP1*/*CCL2*	MCP1-F	GCTATAGAAGAATCACCAGCAGCAA
	MCP1-R	TGGAATCCTGAACCCACTTCTG
*MMP1*	MMP1-F	GTCAGGGGAGATCATCGGG
	MMP1-R	GAGCATCCCCTCCAATACCTG
*MMP3*	MMP3-F	ACAAAGGATACAACAGGGACCAA
	MMP3-R	TAGAGTGGGTACATCAAAGCTTCAGT
*MMP14*	MMP14-F	ACTTTATGGGGGTGAGTCAGG
	MMP14-R	GATGTTGGGCCCATAGGTGG
*NFκBP65*/*RELA*	NFKBP65-F	CGCTTCTTCACACACTGGATTC
	NFKBP65-R	ACTGCCGGGATGGCTTCT
*SPP1*	SPP1-F	CGAGGTGATAGTGTGGTTTATGGA
	SPP1-R	CGTCTGTAGCATCAGGGTACTG
*TGFB1*	TGFB1-F	CACCCGCGTGCTAATGGT
	TGFB1-R	AGAGCAACACGGGTTCAGGTA
*TIMP1*	TIMP1-F	GCCATCGCCGCAGATC
	TIMP1-R	GCTATCAGCCACAGCAACAACA
*TLN1*	TLN1-F	GATGGCTATTACTCAGTACAGACAACTGA
	TLN1-R	CATAGTAGACTCCTCATCTCCTTCCA
*TNFA*	TNFA-F	ATCTTCTCGAACCCCGAGTGA
	TNFA-R	GGCCCGGCGGTTCA
*TUBB*	TUBB-F	CTGGACCGCATCTCTGTGTACTAC
	TUBB-R	GACCTGAGCGAACAGAGTCCAT
*VCL*	VCL-F	GTCTCGGCTGCTCGTATCTT
	VCL-R	GTCCACCAGCCCTGTCATTT
*VEGFA*	VEGFA-F	GCGCTGATAGACATCCATGAAC
	VEGFA-R	CTACCTCCACCATGCCAAGTG
*VIM*	VIM-F	TTCAGAGAGAGGAAGCCGAAAAC
	VIM-R	AGATTCCACTTTGCGTTCAAGGT

All sequences are given in 5′-3′ direction.

### Western blot analysis

Western blotting and densitometry were performed as previously described^41^. The number of investigated samples/per group was n = 5. Equal amounts of 10 µL lysate containing 2 µg/µL protein were loaded and separated on precast TGX stain-free gels (Bio-Rad, Munich, Germany). Transturbo blot PVDF membranes (Bio-Rad) were used for blotting. An overview of the used antibodies and their applied concentrations is given in Table 5. The analysis was performed using ImageQuant™ LAS 4000 (GE Healthcare UK Limited, Buckinghamshire, UK). The membranes were analyzed using ImageJ software (U.S. National Institutes of Health, Bethesda, MD, USA; http://rsb.info.nih.gov/ij/), for densitometric quantification of the bands. Protein from the 3T3 Swiss Albino cell line (American Type Culture Collection, Manassas, VA, USA) was used as a positive control^113^.

**Table 5:** Antibodies applied for Western blot analysis.

Primary Antibody	Company	Dilution	Molecular Weight (kDa)	Catalogue Number
Aggrecan	Abcam	1:1000	210-250	ab36861
α-actin 2	Abcam	1:1000	42	ab5694
β-Actin (HKP)	Sigma	1:1000	42	AC-74
Caveolin-1	Abcam	1:1000	22	ab2910
Collagen I	Abcam	1:500	115-120	ab21286
Collagen IV	Invitrogen	1:1000	160	PA5-50939
CTGF	Sigma	1:1000	38	C4871
E-Cadherin	Abcam	1:1000	120	ab15148
EGF	Sigma	1:1000	26	WH0001950M1
Fibronectin	Invitrogen	1:1000	220	PA5-29578
IL-6	Invitrogen	1:1000	26	AHC0762
IL-8	Abcam	1:1000	11	ab7747
Integrin-β_1_	CST	1:1000	115,135	4706S
JNK1	Abcam	1:1000	48	ab110724
Laminin	Sigma	1:1000	250	L9393
MCP-1/CCL2	Sigma	1:500	18	Mabn712
NF-κB	CST	1:1000	65	C22B4
Osteopontin	Sigma	1:1000	50	o7264
Pan-Actin	CST	1:1000	45	D18C11
Talin	Sigma	1:1000	225-230	T3287
β-Tubulin	Santa Cruz	1:1000	55	sc-5274
VEGF	Abcam	1:1000	23	ab46154
Vimentin	Sigma	1:1000	53	V5255
Vinculin	Sigma	1:1000	116	V9131

The corresponding secondary antibody HRP-linked anti-mouse IgG (Ref. P0260) was used at a dilution of 1:2000, the HRP-linked anti-rabbit IgG (Ref. P0399, both Dako, Carpinteria, CA, USA) was used at a dilution of 1:4000.

### Multianalyte profiling (MAP) technology

Investigation of cellular supernatants for distribution of cytokines and proteins was performed by means of Multianalyte Profiling (MAP) technology. The analysis was carried out using Human Cytokine MAP A v1.0 by Myriad RBM, Inc., Austin, TX, USA. Supernatants of 1*g*-control and RPM-groups where stored at −80 °C and shipped to Myriad RBM.

### Pathway analysis

To investigate and visualize the original localization and the mutual interactions of detected proteins, we entered relevant UniProt accession numbers in a Pathway Studio v.11 software (Elsevier Research Solutions, Amsterdam, The Netherlands)^31,77^.

### Statistical analysis

Statistical Analysis was performed using IBM SPSS Statistics 23.0 software (IBM Deutschland GmbH, Ehningen, Germany) in form of mean ± SD. Statistical deviations were evaluated using Mann-Whitney-U-test. All data is presented as mean ± standard deviation. p < 0.05 was considered as statistically significant.

## Supplementary information

Supplementary Information
