# Supplementary material for: Morphological and Molecular Changes in Juvenile Normal Human Fibroblasts Exposed to Simulated Microgravity
Source: Sci Rep. 2019 Aug 15;9:11882. doi: 10.1038/s41598-019-48378-9 (PMC6695420; doi:10.1038/s41598-019-48378-9)

# MORPHOLOGICAL AND MOLECULAR CHANGES IN JUVENILE NORMAL HUMAN FIBROBLASTS EXPOSED TO SIMULATED MICROGRAVITY

Christoph Buken[a,b], Jayashree Sahana[b], Thomas J. Corydon[b,c], Daniela Melnik[a], Johann Bauer[d], Markus Wehland[a], Marcus Krüger[a], Silke Balk[a], Nauras Abuagela[a], Manfred Infanger[a], Daniela Grimm[a,b,e*]

[a]Clinic and Policlinic for Plastic, Aesthetic and Hand Surgery, Otto-von-Guericke-University, 39120 Magdeburg, Germany

[b]Department for Biomedicine, Aarhus University, Wilhelm Meyers Allé 4, DK-8000 Aarhus C, Denmark

[c]Department of Ophthalmology, Aarhus University Hospital, 8200 Aarhus N, Palle Juul-Jensens Boulevard 99, DK-8200 Aarhus N, Denmark

[d]Max-Planck-Institute of Biochemistry, Martinsried, Am Klopferspitz 18, 82152 Planegg, Germany

[e]Gravitational Biology and Translational Regenerative Medicine, Faculty of Medicine and Mechanical Engineering, Otto-von-Guericke-University Magdeburg, 39120 Magdeburg, Germany

**Supplementary Information**

**Supplementary Figure S1: Full length Western blot panels for Figure 2.**

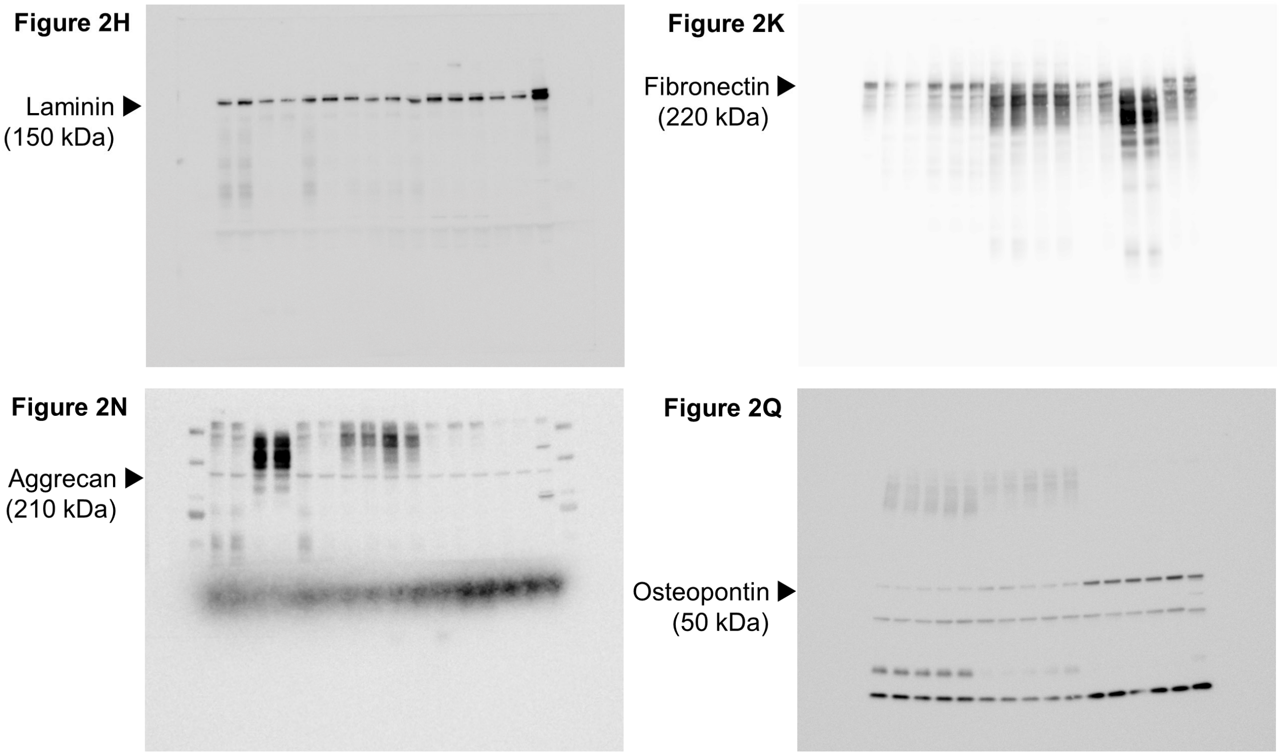

**Supplementary Figure S2: Full length Western blot panels for Figure 3.**

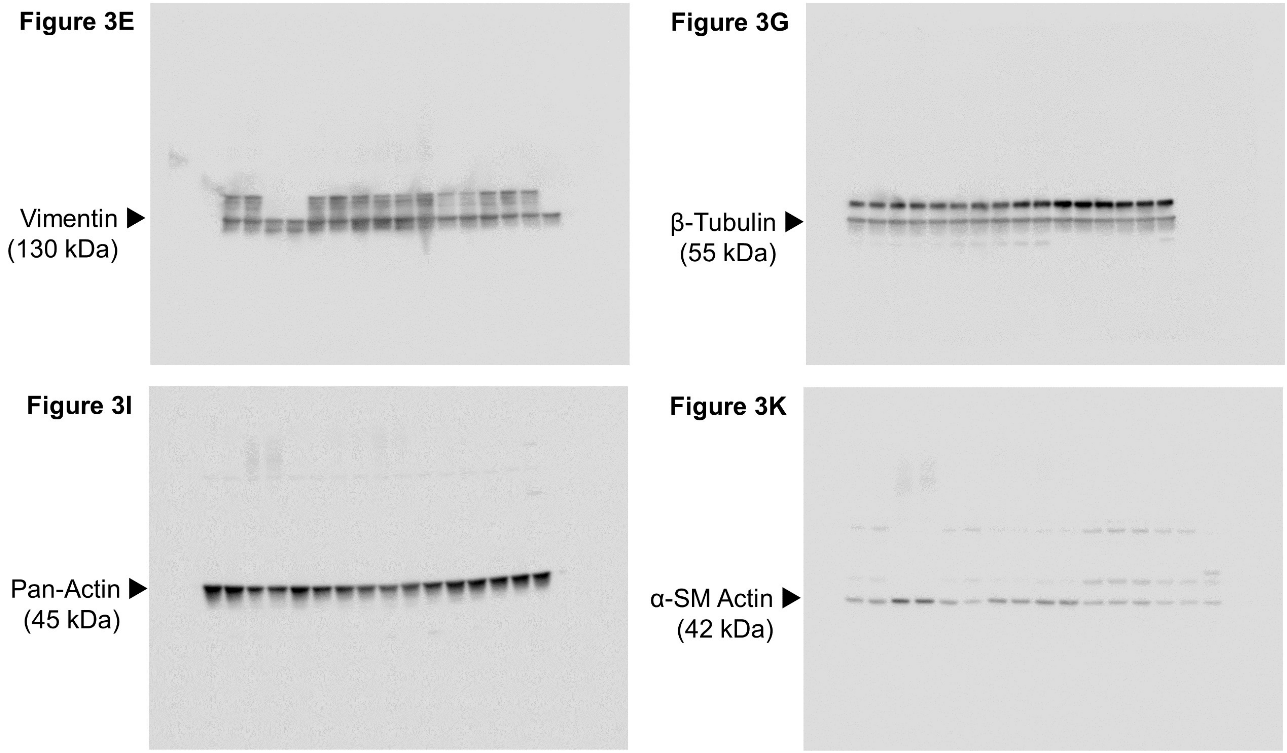

**Supplementary Figure S3: Full length Western blot panels for Figure 4.**

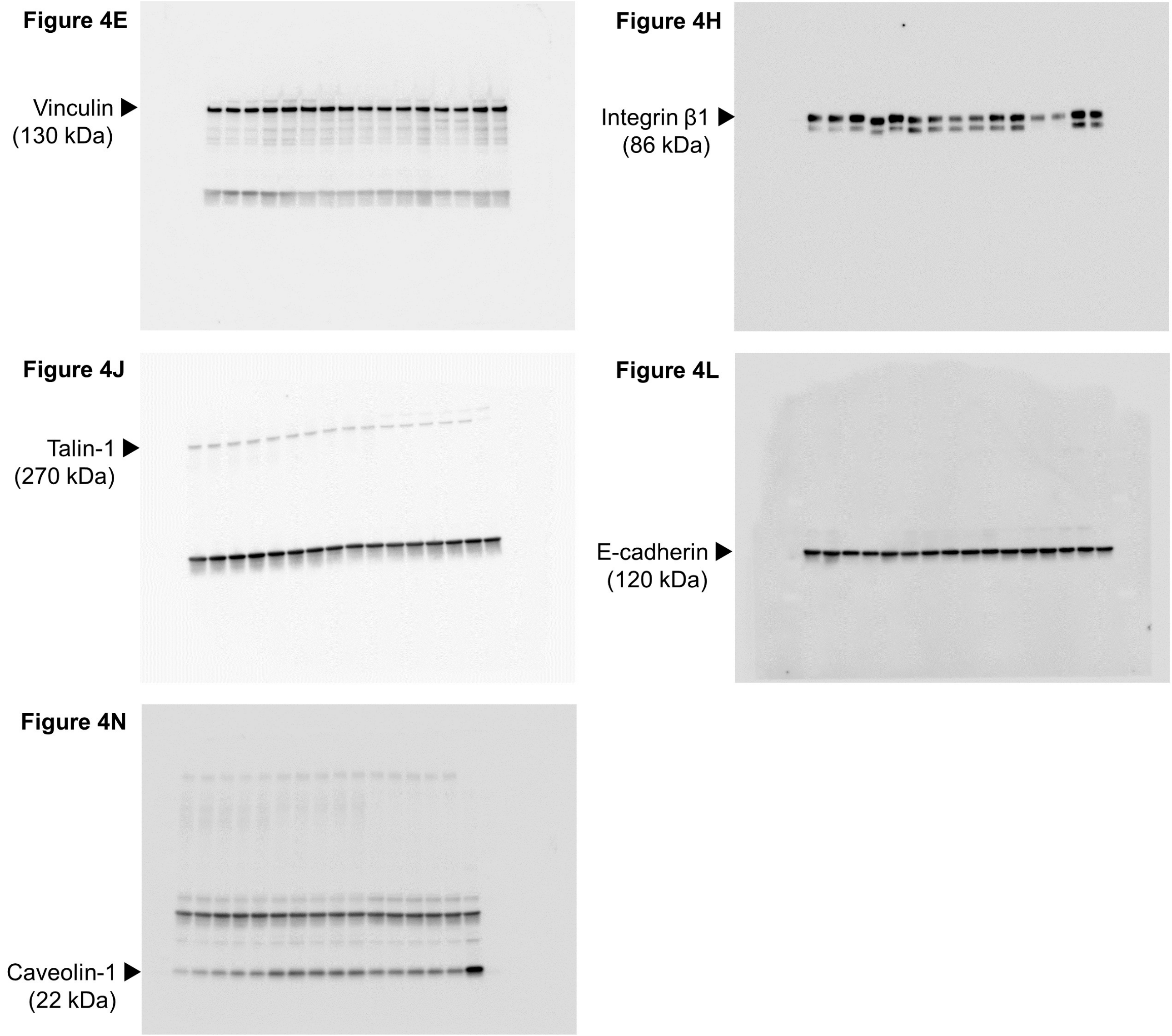

**Supplementary Figure S4: Full length Western blot panels for Figure 5.**

**Figure 5C**

CTGF ▶
(38 kDa)

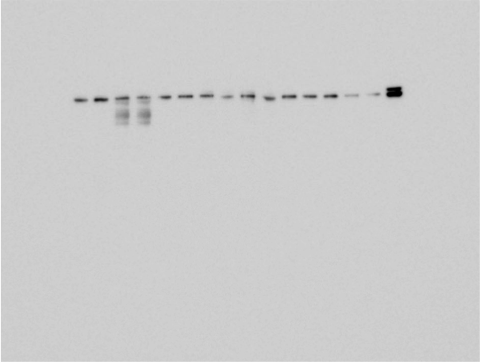

**Figure 5D**

EGF ▶
(26 kDa)

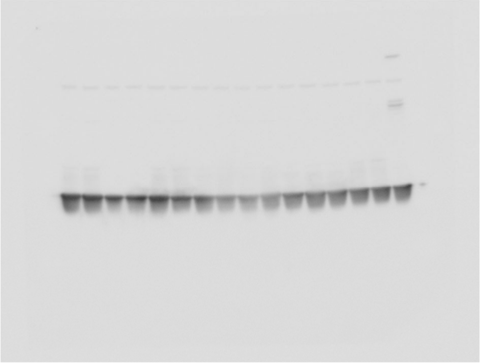

**Figure 5F**

VEGF-A ▶
(23 kDa)

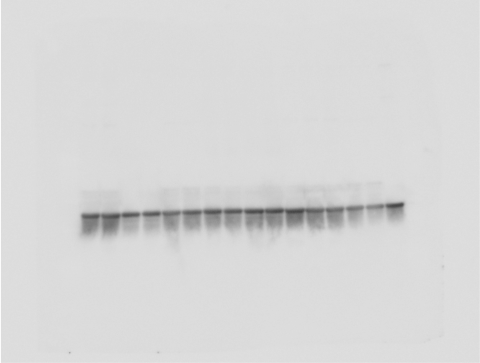

**Supplementary Figure S5: Full length Western blot panels for Figure 6.**

**Figure 6B**

IL-6 ▶
(26 kDa)

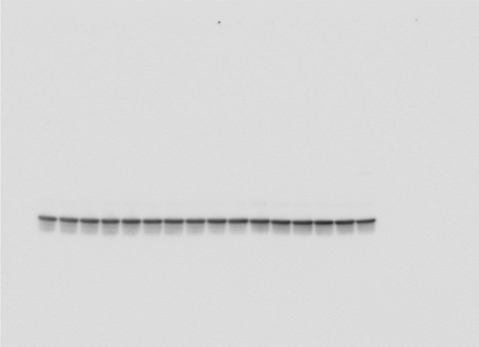

**Figure 6E**

IL-8 ▶
(11 kDa)

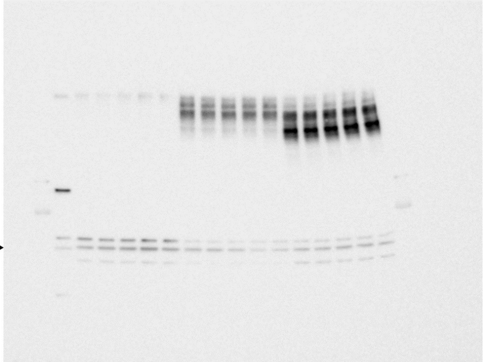

**Figure 6H**

MCP-1 ▶
(18 kDa)

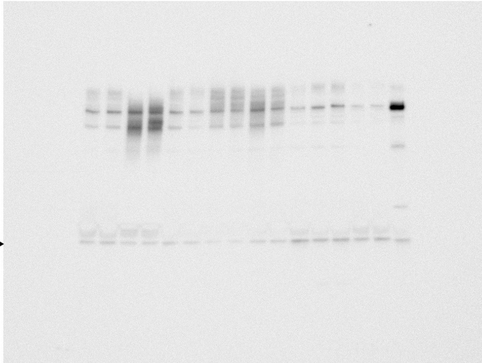

**Figure 6L**

NFκB p65 ▶
(65 kDa)

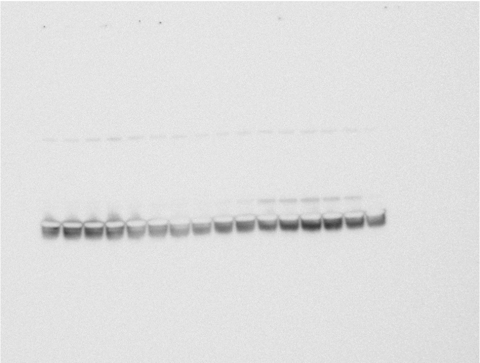

**Figure 6O**

JNK1 ▶
(48 kDa)

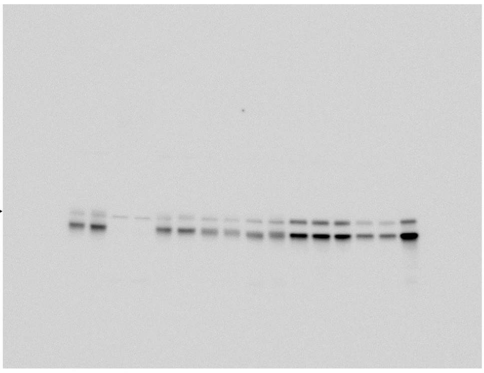

**Supplementary Figure S6: Full length Western blot panels for Figure 7.**

**Figure 7H**

Collagen I ▶
(115 kDa)

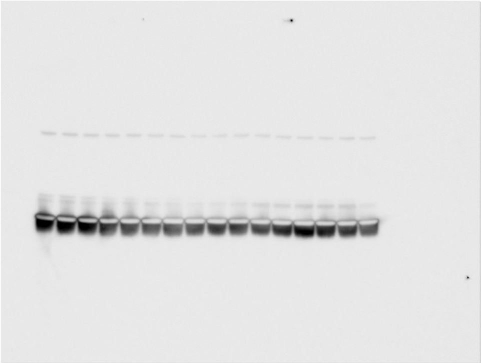

**Figure 7K**

Collagen IV ▶
(160 kDa)

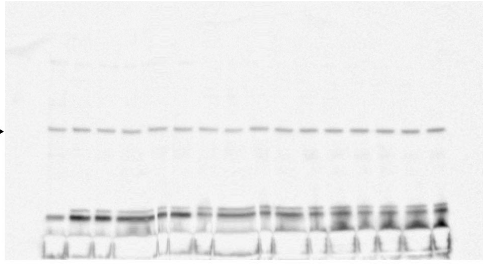

Supplement: Supplementary file 1 — Supplementary Information [file 41598_2019_48378_MOESM1_ESM.pdf]
